# The incubot: A 3D printer-based microscope for long-term live cell imaging within a tissue culture incubator

**DOI:** 10.1016/j.ohx.2021.e00189

**Published:** 2021-03-10

**Authors:** George O.T. Merces, Conor Kennedy, Blanca Lenoci, Emmanuel G. Reynaud, Niamh Burke, Mark Pickering

**Affiliations:** a School of Medicine, University College Dublin, Co. Dublin, D04 V1W8, Ireland; b UCD Centre for Biomedical Engineering, University College Dublin, Co. Dublin, D04 V1W8, Ireland; c School of Biomolecular and Biomedical Science, University College Dublin, Co. Dublin, D04 V1W8, Ireland

**Keywords:** Microscopy, Tissue Culture, Low-Cost, Python, Raspberry Pi, Live-Cell Imaging

## Abstract

Commercial live cell imaging systems represent a large financial burden to research groups, while current open source incubator microscopy systems lack adaptability and are sometimes inadequate for complex imaging experimentation. We present here a low-cost microscope designed for inclusion within a conventional tissue culture incubator. The build is constructed using an entry level 3D printer as the basis for the motion control system, with Raspberry Pi imaging and software integration, allowing for reflected, oblique, and fluorescence imaging of live cell monolayers. The open source nature of the design is aimed to facilitate adaptation by both the community at large and by individual researchers/groups. The development of an adaptable and easy-to-use graphic user interface (GUI) allows for the scientist to be at the core of experimental design through simple modifications of the base GUI code, or generation of an entirely purpose-built script. This adaptability will allow scientists to adapt this equipment for their experimental needs, as opposed to designing experiments to fit their current equipment. The build can be constructed for a cost of roughly €1000 and thus serves as a low-cost and adaptable addition to the open source microscopy community.

## Highlights

• In-incubator microscope can be created using accessible components.
• Graphic user interface allows for simple imaging experiments.
• Accessible design allows for adaptation for more specialised experimentation.
• Oblique and fluorescent illumination modalities.

## Graphical abstract

**Figure:**
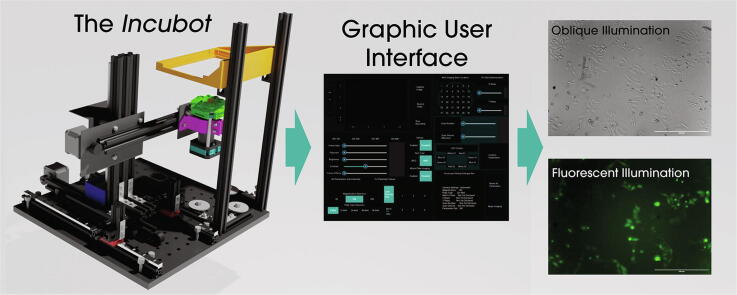

Specifications table

**Table:** 

Hardware name	*Incubot*
Subject area	Biological Sciences (e.g. Microbiology and Biochemistry)
Hardware type	Imaging tools
Open Source License	CC-BY-SA 4.0
Cost of Hardware	~€1000
Source File Repository	https://doi.org/10.17605/OSF.IO/ES3HR

## Hardware in context

Live imaging of cells under physiological conditions is an essential technique in biomedical sciences for analyses of cell proliferation [1], [2], cell migration [3], [4], and cell–cell interactions [5]. Two commercial options are available to researchers aiming to perform live-cell imaging. First, purchase a specific stage incubator designed to upgrade the current expensive microscopy system you already own. Alternatively, purchase a whole new system designed only to be used within a tissue culture incubator, often with a significant price tag, with additional costs for any add-ons such as automated scanning or fluorescence modalities. Despite the widespread use of live cell imaging, the cost of the equipment necessary for such types of experiments remains high. A common way to deal with the significant cost is to arrange a consortium or multi-group effort to purchase the equipment, sharing the resource after purchase, or institutional investment in core imaging facilities. In the case of shared equipment, long-term studies can prove difficult to schedule and can reduce flexibility in experimental design, particularly in early stage, exploratory, and scoping studies. Usage fees applied to maintain the equipment may also then limit access to lower income laboratories. This cost barrier to owning complex imaging equipment can stifle research efforts in the biomedical field. Low-cost alternatives have recently been added to the market, notably the Etaluma series of incubator microscopes. Products such as these partially fill the gap in the market, with high quality imaging capabilities and options for automation through their SDKs. However, any commercial solution has key drawbacks, mainly centred on the lack of flexibility around hardware and software, along with substantially higher costs (£45,000-£63,000 GBP for the Etaluma LS720 (information gained from a UK supplier quote)). With a commercial option, the user must rely on their specific LEDs and filters (and thus the specific wavelengths associated with them), their specific camera, and usually their user interface.

The open source movement within science has been providing innovations in affordable laboratory equipment, including microscopy and optics [6], [7], [8], [9]. Open source approaches for low-cost incubator microscopes have been explored [10], [11], [12], [13] and serve as great examples of affordable microscopes for live-cell imaging. Specific examples such as that created by Schneidereit *et al* [14] provide a cheap solution for high-content screening microscopy within an incubator, however its design for a single purpose makes adaptation for other experimental processes difficult. Designs such as work by Wincott *et al* [15] offer adaptability along with a low-cost, however imaging quality may not be sufficient for a large range of research applications. Additionally, work by Katunin *et al* [16] bridges these concerns, allowing for adaptability and reasonable imaging quality, but for a substantially higher price if fluorescence imaging is desired. General limitations with other examples of current designs curtail their potential widespread use within laboratories. The use of a CNC machine is neither common nor accessible amongst biomedical researchers. Options that allow for in-incubator microscopy but lack a motion control system result in a low-throughput. The use of only transmitted white light reduces the range of potential applications of other builds. An ideal scenario would be an affordable open source system that makes use of conventional low-cost commercial products, achieving a high imaging quality, while allowing for adaptability as a group’s research interests or capabilities may change.

Microscopy is always a trade-off between field of view (FOV) and resolution. A static microscope is simple and effective, but only allows a small FOV to be imaged. Imaging more than a single FOV requires some element of motion control of either the optics or the sample. The open flexure microscope is a remarkable solution to this problem, as it includes very precise motion control at a low cost [17]. The main drawback is that the range of movement is limited and does not allow for the scanning of plates or flasks that are routinely used in cell culture experiments. Luckily, the problem of precise and repeatable control of three-dimensional movement has been solved in another field: 3D printing. Commercial 3D printers require sub-millimetre precision over a range of travel of tens of centimetres. Therefore, we designed an incubator microscope, the *Incubot*, which repurposes the core motion control system from a simple, entry-level cartesian 3D printer (a Tronxy X1). An associated user guide has been made available (https://osf.io/ht24j/) to help explain all steps in construction of this system, with the aim that non-experts in microscopy or optics can construct and use this equipment.

## Hardware description

**Fig. 1:**
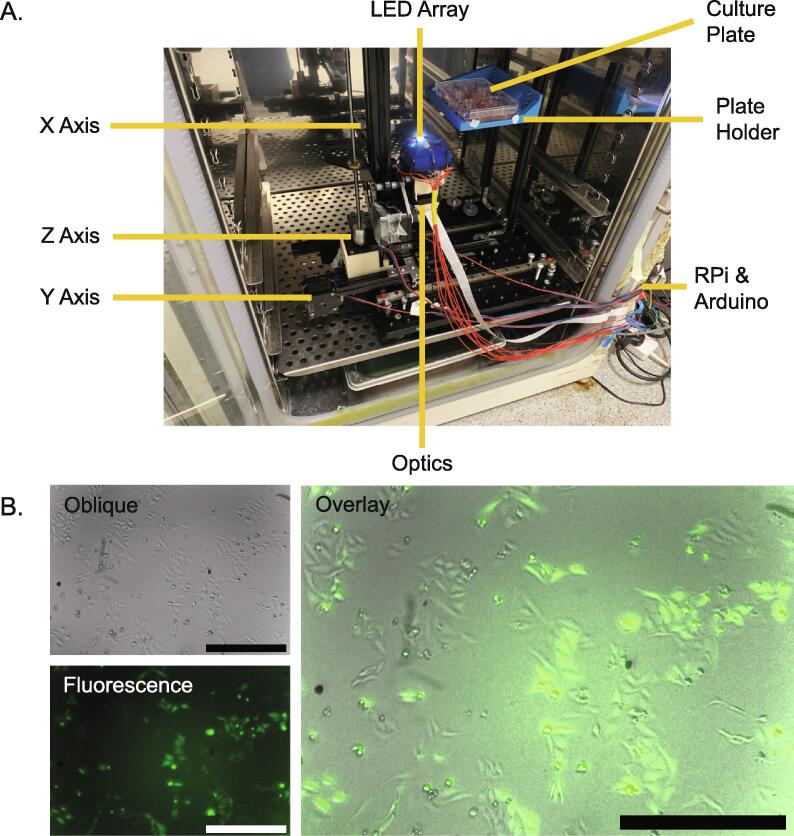
A) Fully assembled Incubot within a tissue culture incubator with key components/features annotated. B) Representative images of HeLa-GFP live cells within an incubator using the Incubot using oblique white illumination (Oblique), blue LED excitation of GFP (Fluorescence) and the two images overlaid (Overlay). Scale bars indicate 500 µm. (For interpretation of the references to colour in this figure legend, the reader is referred to the web version of this article.)

The *Incubot* uses a Tronxy X1 3D printer as a motorised stage for optics movement allowing for XYZ imaging of tissue culture flasks or plates (Fig. 1.). The X and Z axes are coupled directly to the Y axis to allow for the 3-dimensional movement of the optics unit which is coupled to the mount point that originally supported the extruder of the printer. A tissue culture plate is positioned above the optics via a stage composed of 15x15 mm aluminium extrusion and 3D-printed components, which remains stationary while the optics are translated. Moving the optics while keeping the sample static prevents movement of the medium during imaging, which may affect the cells. The optics unit is composed of 1″ ø tubes, connecting a commercial infinite-conjugate objective lens to a Raspberry Pi Camera (V2), using an achromatic doublet tube lens to allow for microscopic image acquisition. The optional inclusion of a long pass filter allows for fluorescent imaging of samples. A silicone moulded rig was used to mount 8 LEDs around the objective lens, and thus allow for several illumination techniques to be performed, such as reflected, oblique, and fluorescent. Both the motorised stage and the plate-holder are mounted onto a 300 mm × 300 mm breadboard (M6) with vibration-absorbing feet. The whole unit can be placed within a tissue culture incubator for long term use, allowing for inverted imaging of a tissue culture plate/flask within an incubator maintained at physiological conditions. As the entire system is maintained at 37 °C it avoids the negative impact of thermal drift associated with temperature gradients present in heated stages.

A Graphical User Interface (GUI) was developed using Kivy 2.0.0 for Python 3, allowing for simple user control over parameters for live cell imaging, including the number of time points to image over, the temporal spacing between them, the number and layout of wells, the area of each well imaged, and automated focusing for each well. The total cost of this system (~€1000) represents a substantial saving over commercial equipment [18]. We have established an additional repository on GitHub (https://github.com/GeorgeMerces/Incubot/upload/main) for greater interactivity between us and potential users for distribution of improvements.

This build represents a low-cost alternative to commercial microscopy systems for physiological imaging of cells, with specific utility in simple imaging experimentations. The use of simple components and 3D printing for construction of the build allow for modification and adaptation of the build based on individual user needs.

Potential uses of the *Incubot*:

• Long-term monitoring of tissue culture experiments
• Long-term monitoring of tissue culture flasks for determination of optimum time for passaging/experimentation
• Semi-portable device for tissue culture information outreach
• Use as a regular scanning microscope for fixed cell imaging

**Table:** 

***Incubot* Parameter**	**Details**
*Incubot* Dimensions (mm)	300 × 370 × 370
Sensor Resolution (Raspberry Pi Camera V2, Pixels)	*Maximum: 3280 × 2464*, *Incubot Default: 1680x1200*
Max Movement Speed (Tronxy X1, [X, Y], mm/s)	*[130, 130]*
Movement Tolerance [X, Y]	0.1-1.0 mm Range 5%, Greater than 1.0 mm Range = median error of ± 13 µ*m*
Field of View Dimensions (10X Objective, [X, Y] µm)	[1300 x 925]
Maximum Pixel Resolution (10X Objective, default Incubot pixel resolution of 1680x1200 pixels)	*1 pixel =*0.855 µ*m^2^, Theoretical Resolution Limit = 982 lines.mm^−1^*, Measured Resolution Limit = ≥228.1 lines.mm^−1^
Imaging Run Time (24 Well Plate, 4x4 [16] Images per Well, *Incubot* Resolution)	*BMP = 32.8 Minutes, JPEG = 7.2 Minutes*
Image Storage Size	*BMP = 5.76 MB, JPEG = 1.16 MB*

## Design files

Design Files Summary

**Table:** 

**Design file name**	**File type**	**Open source license**	**Location of the file**
XYZ_Coupler	3D Model (.stl)	CC-BY-SA 4.0	https://doi.org/10.17605/OSF.IO/ES3HR
Optics_Holder	3D Model (.stl)	CC-BY-SA 4.0	https://doi.org/10.17605/OSF.IO/ES3HR
Pi_Camera_Holder	3D Model (.stl)	CC-BY-SA 4.0	https://doi.org/10.17605/OSF.IO/ES3HR
LED_Holder_Mould	3D Model (.stl)	CC-BY-SA 4.0	https://doi.org/10.17605/OSF.IO/ES3HR
3D_Printed_LED_Holder	3D Model (.stl)	CC-BY-SA 4.0	https://doi.org/10.17605/OSF.IO/ES3HR
Plate_Holder	3D Model (.stl)	CC-BY-SA 4.0	https://doi.org/10.17605/OSF.IO/ES3HR
CameraPreview	Python Script (.py)	CC-BY-SA 4.0	https://doi.org/10.17605/OSF.IO/ES3HR
MotionValidationProtocol	Python Script (.py)	CC-BY-SA 4.0	https://doi.org/10.17605/OSF.IO/ES3HR
IncubotGUI	Python Script (.py)	CC-BY-SA 4.0	https://doi.org/10.17605/OSF.IO/ES3HR
StationaryStabilityTesting	Python Script (.py)	CC-BY-SA 4.0	https://doi.org/10.17605/OSF.IO/ES3HR
MotionRestTesting	Python Script (.py)	CC-BY-SA 4.0	https://doi.org/10.17605/OSF.IO/ES3HR
PairwiseStitchingMotionValidation	ImageJ Macro (.ijm)	CC-BY-SA 4.0	https://doi.org/10.17605/OSF.IO/ES3HR
Movement Validation Template	Excel Sheet (.xlsx)	CC-BY-SA 4.0	https://doi.org/10.17605/OSF.IO/ES3HR
Motion Validation GUI	Python Script (.py)	CC-BY-SA 4.0	https://doi.org/10.17605/OSF.IO/ES3HR
Well Location GUI	Python Script (.py)	CC-BY-SA 4.0	https://doi.org/10.17605/OSF.IO/ES3HR

XYZ_Coupler: 3D printed unit designed to couple the X and Z axes of the original Tronxy X1 to its Y axis.

Optics_Holder: 3D printed unit to replace the extruder portion of the original Tronxy X1. Used to clamp hold of the optics and to couple XYZ motion to the camera.

Pi_Camera_Holder: 3D printed unit to hold the Raspberry Pi Camera in the correct orientation and distance from the lenses of the optical system.

LED_Holder_Mouldhttps://doi.org/10.17605/OSF.IO/ES3HR: 3D printed negative mould for casting a silicone LED-holding ring.

Plate_Holder: 3D printed unit to allow for stable elevation of a tissue culture plate over the optics of the *Incubot* for imaging.

MotionValidationProtocol: Python script to perform sequential positive and negative movements of known distance in the X and Y axes independently. Images are acquired after each movement to allow for later confirmation of movement distances.

IncubotGUI: Python script that uses Kivy to generate a graphical user interface for interacting with the *Incubot* and performing imaging experiments.

StationaryStabilityTesting: Python script to perform repeat imaging of a stationary location over time for XY drift tracking.

MotionRestTesting: Python script to determine the necessary time between movement commands and image acquisition for optimum *Incubot* function.

PairwiseStitchingMotionValidation: ImageJ Macro to stitch sequential images from the MotionValidationProtocol.py script for analysis of motion distances moved.

Movement Validation Template: Excel file template to input data resulting from the PairwiseStitchingMotionValidation.ijm ImageJ macro.

Motion Validation GUI: Python GUI script to guide the user through the process of calibrating their *Incubot* to move correctly in response to motion commands.

Well Location GUI: Python GUI script to guide users through the calibration process for specifying the location of key well landmarks necessary for defining the region where a tissue culture plate will be present.

## Bill of materials

Please note that suppliers mentioned here are just suggestions and are in no way endorsements of the sites/products themselves. Material costs may vary over time and depending on location, and alternative sources may provide better prices than those listed here. We encourage readers building this design to identify their own sources for materials suitable for their location and needs. In addition to this list, we have included a list using institutionally approved suppliers, based on our own institution (https://doi.org/10.17605/OSF.IO/ES3HR). Please note that three items were unable to be found from approved vendors, these items being the 3D printer itself, the CNC motor shield, and the Drv8825 stepper motor drivers.

**Table:** 

**Designator**	**Component**	**Number**	**Cost per unit – (€)**	**Total cost - (€)**	**Source of materials**	**Material type**
**General Components (MakerBeam)**						
**MBXL300**	300 mm black MakerBeamXL	4	3.25	13	https://www.makerbeam.com/300mm-4p-black-makerbeamxl-15mmx15mm.html	Metal
**MBXL200**	200 mm black MakerBeamXL	1	2.31	2.31	https://www.makerbeam.com/200mm-4p-black-makerbeamxl-15mmx15mm.html	Metal
**MBXL150**	150 mm black MakerBeamXL	1	1.75	1.75	https://www.makerbeam.com/150mm-4p-black-makerbeamxl-15mmx15mm.html	Metal
**MBXL100**	100 mm black MakerBeamXL	1	1.25	1.25	https://www.makerbeam.com/100mm-4p-black-makerbeamxl-15mmx15mm.html	Metal
**MBXL50**	50 mm black MakerBeamXL	7	0.63	4.38	https://www.makerbeam.com/50mm-4p-black-makerbeamxl-15mmx15mm.html	Metal
**MBR**	30 cm M3 Compatible Linear Rails with Carriage (MakerBeam)	2	21.5	43	https://www.makerbeam.com/300mm-linear-slide-rail-and-carriage.html	Metal
**M3S25**	Square Headed Bolts 25 mm (25p) for MakerBeam	6	0.08	0.47	https://www.makerbeam.com/makerbeam-square-headed-bolts-25mm-25p-for-makerbe.html	Metal
**M3S12**	Square Headed Bolts 12 mm (100p) for MakerBeam	9	0.08	0.7317	https://www.makerbeam.com/makerbeam-square-headed-bolts-12mm-100p-for-makerb.html	Metal
**M3S6**	Square Headed Bolts 6 mm (250p) for MakerBeam	68	0.06	3.77	https://www.makerbeam.com/makerbeam-square-headed-bolts-6mm-250p-for-makerbe.html	Metal
**M3N**	Nuts Regular (250p)	73	0.02	1.31	https://www.makerbeam.com/makerbeam-nuts-regular-250p.html	Metal
**MBCB**	MakerBeam Corner brackets (MakerBeamXL and OpenBeam compatible)	6	0.58	3.47	https://www.makerbeam.com/makerbeam-corner-brackets-12p.html	Metal
**MBXLLB**	MakerBeam XL Right Angle Bracket (12p)	4	0.75	2.98	https://www.makerbeam.com/makerbeam-makerbeam-xl-right-angle-bracket-12p.html	Metal
**MBXLTB**	MakerBeam XL T Bracket (12p)	2	0.75	1.49	https://www.makerbeam.com/makerbeam-makerbeam-xl-t-bracket-12p.html	Metal
**M3NT**	T-Slot Nuts for MakerBeam (25p)	4	0.2	0.8	https://www.makerbeam.com/t-slot-nuts-for-makerbeamxl-50p.html	Metal
**MBXLCC**	Corner Cubes Black (12p) − 15mmx15mmx15mm	6	1.25	7.47	https://www.makerbeam.com/openbeam-corner-cubes-black-12p-for-makerbeamxl-or.html	Metal
**General Components (Additional)**						
**M6S25**	Bright Zinc Plated Steel Hex Bolt, M6 × 25 mm	16	0.25	4	https://ie.rs-online.com/web/p/hex-bolts/9173107/	Metal
**M5S50**	RS PRO Pozidriv Pan Head Bright Zinc Plated Steel Machine Screw, M5, 50 mm	3	0.17	0.5	https://ie.rs-online.com/web/p/machine-screws/9087719?cm_mmc=IE-PLA-DS3A-_-google-_-PLA_IE_EN_Fasteners_And_Fixings-_-Screws_And_Bolts%7CMachine_Screws-_-PRODUCT_GROUP&amp;matchtype=&amp;pla-394349412895&amp;gclid=EAIaIQobChMIy_OiiuW35wIVBLTtCh1KJA6REAQYAyABEgJI3vD_BwE&amp;gclsrc=aw.ds	Metal
**3D Printed Components**						
**3DWS**	3D-Printed Wheel Spacer, PLA, 0.15 mm Resolution, 25% Infill	1	0.4	0.4	https://www.thingiverse.com/thing:2203872	Polymer
**3DXYZC**	3D-Printed XYZ Coupler, PLA, 0.15 mm Resolution, 25% Infill	1	1.69	1.69	https://doi.org/10.17605/OSF.IO/ES3HR	Polymer
**3DOH**	3D-Printed Optics Holder, PLA, 0.15 mm Resolution, 25% Infill	1	1.05	1.05	https://doi.org/10.17605/OSF.IO/ES3HR	Polymer
**PiCH**	3D-Printed Pi Camera Holder, PLA, 0.15 mm Resolution, 15% infill	1	0.51	0.51	https://doi.org/10.17605/OSF.IO/ES3HR	Polymer
**LEDHM**	3D-Printed LED Holder Mould, PLA, 0.15 mm Resolution, 15% infill	1	6.31	6.31	https://doi.org/10.17605/OSF.IO/ES3HR	Polymer
**3DPH**	3D-Printed Plate Holder, PLA, 0.15 mm Resolution, 15% infill	1	4.71	4.71	https://doi.org/10.17605/OSF.IO/ES3HR	Polymer
**Motion Control Hardware**						
**X1P**	Tronxy X1 3D Printer Printing Machine Educational Desktop Print 3D with 150x150x150mm - X1 (Gearbest)	1	135.84	135.84	https://www.gearbest.com/3d-printers-3d-printer-kits/pp_494194.html?wid=1433363&currency = EUR&vip = 4265218&gclid = EAIaIQobChMIluiuttiR5wIVQ7TtCh2BYAQGEAQYASABEgIMqPD_BwE	Other
**BB**	Thorlabs - MB3030/M - Aluminium Breadboard, 300 mm × 300 mm × 12.7 mm, M6 Taps	1	141.38	141.38	https://www.thorlabs.com/thorproduct.cfm?partnumber=MB3030/M	Metal
**AUR3**	Arduino Uno R3 USB Microcontroller	1	21.49	21.49	https://www.robotshop.com/eu/en/arduino-uno-r3-usb-microcontroller.html?gclid=EAIaIQobChMIufmc4bzB5gIVh6ztCh0yaQzNEAQYAiABEgK-BfD_BwE	Other
**CNCMS**	KINGPRINT CNC Shield V3.0 Expansion Board for Arduino with 4pcs A4988 Stepper Motor Driver with Heatsink kits for Arduino	1	14.11	14.11	https://www.amazon.co.uk/KINGPRINT-Expansion-Arduino-Stepper-Heatsink/dp/B0799JLFV4/ref=asc_df_B0799JLFV4/?tag=googshopuk-21&linkCode=df0&hvadid=310830238305&hvpos=1o2&hvnetw=g&hvrand=4202945403292490884&hvpone=&hvptwo=&hvqmt=&hvdev=c&hvdvcmdl=&hvlocint=&hvlocphy=9040157&hvtargid=pla-563353773270&psc=1	Other
**SMD**	Drv8825 Stepper Motor Driver	3	2.75	8.24	https://www.amazon.co.uk/ANGEEK-Drv8825-Stepper-Driver-Printer/dp/B07WRGTC58/ref=sr_1_3_sspa?keywords=DRV8825&amp;qid=1581003378&amp;sr=8%E2%80%933-spons&amp;ps=1&amp;spLa=ZW5jcnlwdGVkUXVhbGlmaWVyPUFIUVBJNzNQTDU3QU4mZW5jcnlwdGVkSWQ9QTA2OTAzOTgyOUhEUUNGMFhOTklIJmVuY3J5cHRlZEFkSWQ9QTAyNzQyNzQxM0RDTEVZNzA3Q1NLJndpZGdldE5hbWU9c3BfYXRmJmFjdGlvbj1jbGlja1JlZGlyZWN0JmRvTm90TG9nQ2xpY2s9dHJ1ZQ==	Other
**USB**	30CM Blue USB 2.0 Type A Male to Type B Male Power Data Transmission Cable for UNO R3 MEGA 2560	1	1.52	1.52	https://uk.banggood.com/30CM-Blue-USB-2_0-Type-A-Male-to-Type-B-Male-Power-Data-Transmission-Cable-For-Arduino-p-1306749.html?gmcCountry=IE&amp;currency=EUR&amp;createTmp=1&amp;utm_source=googleshopping&amp;utm_medium=cpc_bgcs&amp;utm_content=zouzou&amp;utm_campaign=pla-ieg-ele-diy1-pc&amp;ad_id=337459540786&amp;gclid=EAIaIQobChMI68n9kL7B5gIVRrTtCh0djwomEAQYAiABEgL-FvD_BwE&amp;cur_warehouse=CN	Other
**Optics**						
**PiCV2**	Raspberry Pi Camera V2 Camera Module, CSI-2, 3280 × 2464 Resolution	1	27.5	27.5	https://ie.rs-online.com/web/p/video-modules/9132664/	Other
**RCL**	Adafruit Flex Cable for Raspberry Pi Camera − 24″ / 610 mm [ADA1731]	1	3.1	3.1	https://www.amazon.co.uk/Adafruit-Flex-Cable-Raspberry-Camera/dp/B00M4DAQH8/ref	Other
**SM1L10**	SM1L10 - SM1 Lens Tube, 1.00″ Thread Depth, One Retaining Ring Included	1	13.34	13.34	https://www.thorlabs.com/thorproduct.cfm?partnumber=SM1L10	Metal
**SM1L05**	SM1L05 - SM1 Lens Tube, 0.50″ Thread Depth, One Retaining Ring Included	1	11.79	11.79	https://www.thorlabs.com/thorproduct.cfm?partnumber=SM1L05	Metal
**OBJL**	Olympus PLN 10X Objective	1	315	315	https://www.edmundoptics.eu/p/olympus-pln-10x-objective/29222/	Metal
**SRA**	SM1A3 - Adapter with External SM1 Threads and Internal RMS Threads	1	16.82	16.82	https://www.thorlabs.com/thorproduct.cfm?partnumber=SM1A3	Metal
**TLCP**	CP33/M - SM1-Threaded 30 mm Cage Plate, 0.35″ Thick, 2 Retaining Rings, M4 Tap	3	15.35	46.05	https://www.thorlabs.com/thorproduct.cfm?partnumber=CP33/M	Metal
**CAR3**	Cage Assembly Rod, 3″ Long, Ø6 mm	1	5.87	5.87	https://www.thorlabs.com/thorproduct.cfm?partnumber=ER3-P4	Metal
**CAR1**	Cage Assembly Rod, 1″ Long, Ø6 mm	2	4.49	9.98	https://www.thorlabs.com/thorproduct.cfm?partnumber=ER1-P4	Metal
**TL**	AC254-050-A - f = 50.0 mm, Ø1″ Achromatic Doublet, ARC: 400–700 nm	1	73.77	73.77	https://www.thorlabs.com/thorproduct.cfm?partnumber=AC254-050-A	Other
**LPF**	SCHOTT OG-515, 25.4 mm Dia., Long pass Filter	1	33.19	33.19	https://www.edmundoptics.com/p/og-515%E2%80%9325.4mm-dia.-longpass-filter/5560/	Other
**DSSil**	50 g Silicone (Dragon Skin™ 10 Medium) Cast to LEDHM and Cured	1	2.13	2.13	https://www.benam.co.uk/products/silicone/addition/dragon-skin/dragon-skin-10-medium	Polymer
**LEDw, LEDb, LEDr, LEDg**	ATPWONZ 300pcs 3 mm 5 mm 2pin Light Emitting Diodes Round Head LED Lamp Assorted Colour Diodes Resistor Kit	1	5.94	0.1584	https://www.amazon.co.uk/ATPWONZ-300pcs-Emitting-Assorted-Resistor/dp/B06X3VT6TD/ref=asc_df_B06X3VT6TD/?tag=googshopuk-21&amp;linkCode=df0&amp;hvadid=231995364607&amp;hvpos=1o2&amp;hvnetw=g&amp;hvrand=6766138935065738399&amp;hvpone=&amp;hvptwo=&amp;hvqmt=&amp;hvdev=c&amp;hvdvcmdl=&amp;hvlocint=&amp;hvlocphy=9040157&amp;hvtargid=pla-348541696479&amp;psc=1	Other
**Additional Electronics**						
**Pi3B+**	Raspberry Pi 3 Model B + SBC Computer Board	1	38.41	38.41	https://ie.rs-online.com/web/p/processor-microcontroller-development-kits/1373331?cm_mmc=IE-PLA-DS3A-_-google-_-PLA_IE_EN_Semiconductors-_-Semiconductor_Development_Kits%7CProcessor_And_Microcontroller_Development_Kits-_-PRODUCT_GROUP&amp;matchtype=&amp;pla-393439849165&amp;amp;gclid=EAIaIQobChMI4PWHpb7B5gIVjbHtCh3uCwnLEAQYASABEgJla_D_BwE&amp;amp;gclsrc=aw.ds	Other
**Pi3B + POW**	Raspberry Pi Raspberry Pi Power Supply, Micro USB Type B with Universal Plug Type, 1.5 *m*	1	8.66	8,66	https://ie.rs-online.com/web/p/raspberry-pi-power-supplies/9098126/	Other
**mSDC**	Integral Micro SDHC Card V10 32 GB	1	5.89	5.89	https://www.vikingdirect.ie/en/integral-sdhc-card-v10-32-gb-p-1015415?cm_mmc=Google-_-PLA_GEN_GOOGLE-SHOPPING_technology_GOSC-_-technology-_-1015415&amp;gclid=EAIaIQobChMIovLxr7_B5gIVhbTtCh3jNAk1EAQYBSABEgKuQPD_BwE&amp;gclsrc=aw.ds	Other

## Build instructions

### General Safety Notice

The build will ultimately require a 12 V power supply to the CNC motor shield, and 5 V power supply to the Raspberry Pi. Take care when performing any soldering to prevent short circuit generation or injury while working with intense heat.

### General Assembly Notice

There are many connections listed below that rely on a M3 screw and a M3 nut. Please note that in most cases it is possible to reverse the orientation of these while constructing the *Incubot*, with no negative impact on build stability or quality. If you find inserting the screws into a MakerBeam groove to be easier during construction than sliding the nut, then please do whatever is easiest for you.

An animated assembly overview can be found within the online repository (https://doi.org/10.17605/OSF.IO/ES3HR).

**Fig. 2:**
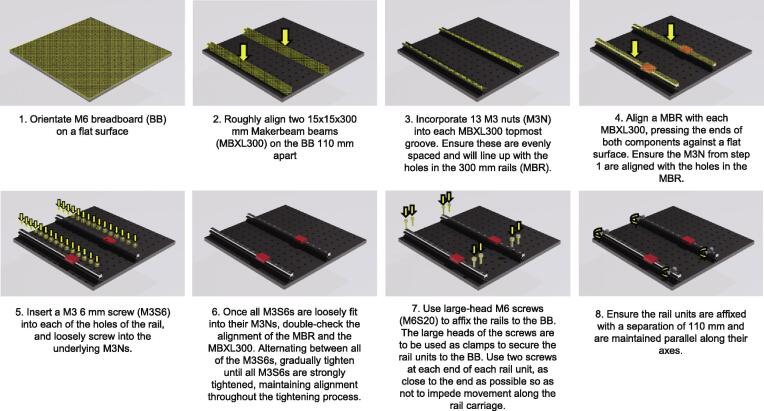
Incubot Assembly Visual Guide – Rail Assembly.

Rail Assembly (Fig. 2.)

1. Orientate the M6 breadboard (BB) on a flat surface.
2. Roughly align two 15x15x300 mm MakerBeam beams (MBXL300) on the BB 110 mm apart.
3. Incorporate 13 M3 nuts (M3N) into each MBXL300 topmost groove. Ensure these are evenly spaced and will line up with the holes in the 300 mm rails (MBR).
4. Align an MBR with each MBXL300, pressing the ends of both components against a flat surface. Ensure the M3N from step 1 are aligned with the holes in the MBR.
5. Insert a M3 6 mm screw (M3S6) into each of the holes of the rail, and loosely screw into the underlying M3Ns.
6. Once all M3S6s are loosely fit into their M3Ns, double-check the alignment of the MBR and the MBXL300. Alternating between all the M3S6s, gradually tighten until all M3S6s are strongly tightened, maintaining alignment throughout the tightening process.
  a. NOTE: Aligning the MBR and the MBXL300 at the ends can be done by pressing the ends of both against a flat object and maintaining pressure during the screw tightening process.
7. Use large-head M6 screws (M6S20) to affix the rails to the BB. The large heads of the screws are to be used as clamps to secure the rail units to the BB. Use two screws at each end of each rail unit, as close to the end as possible so as not to impede movement along the rail carriage.
8. Ensure the rail units are affixed with a separation of 110 mm and are maintained parallel along their axes.

**Fig. 3:**
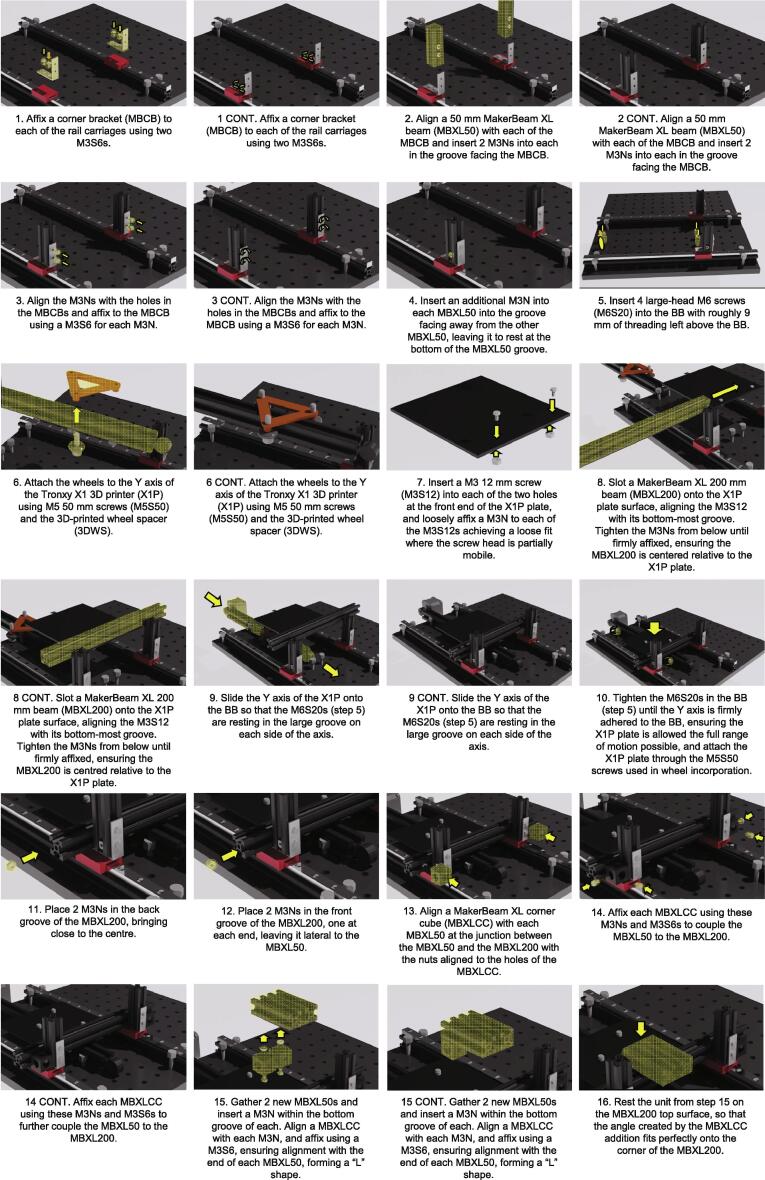
Incubot Assembly Visual Guide – Axes Coupling Part I.

**Fig. 4:**
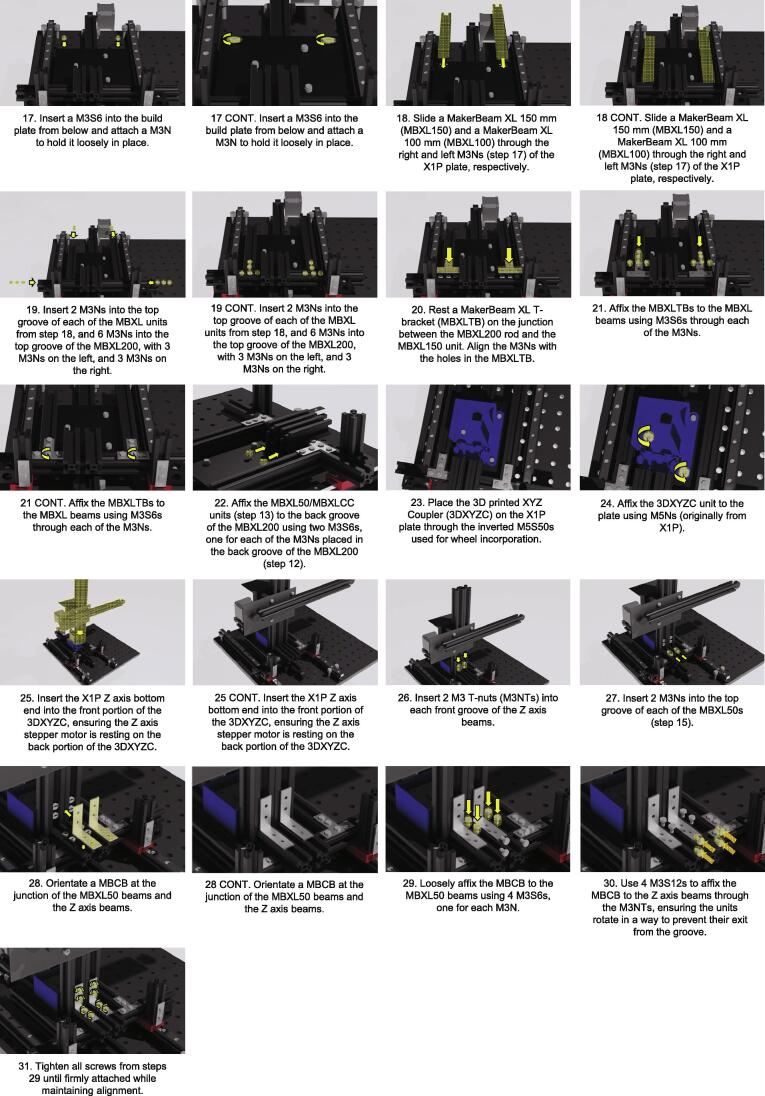
Incubot Assembly Visual Guide – Axes Coupling Part II.

Coupling the X, Y and Z axes (Fig. 3. Fig. 4.)

1. Affix a corner bracket (MBCB) to each of the rail carriages using two M3S6s.
2. Align a 50 mm MakerBeam XL beam (MBXL50) with each of the MBCB and insert 2 M3Ns into each in the groove facing the MBCB.
3. Align the M3Ns with the holes in the MBCBs and affix to the MBCB using a M3S6 for each M3N.
4. Insert an additional M3N into each MBXL50 into the groove facing away from the other MBXL50, leaving it to rest at the bottom of the MBXL50 groove.
5. Insert 4 large-head M6 screws (M6S20) into the BB with roughly 9 mm of threading left above the BB.
  a. NOTE: Orientate these screws so that there is one pair in the very back row of BB holes, and the other pair is roughly 230 mm away. The screws within each pair should be in adjacent BB holes.
6. Attach the wheels to the Y axis of the Tronxy X1 3D printer (X1P) using M5 50 mm screws (M5S50) and the 3D-printed wheel spacer (3DWS).
  a. NOTE: This unit will not be firmly adhered as the nuts (M5Ns, originally from X1P) will not be attached until later, so be careful when moving this unit until then.
7. Insert a M3 12 mm screw (M3S12) into each of the two holes at the front end of the X1P plate, and loosely affix a M3N to each of the M3S12s achieving a loose fit where the screw head is partially mobile.
8. Slot a MakerBeam XL 200 mm beam (MBXL200) onto the X1P plate surface, aligning the M3S12 with its bottom-most groove. Tighten the M3Ns from below until firmly affixed, ensuring the MBXL200 is centred relative to the X1P plate.
9. Slide the Y axis of the X1P onto the BB so that the M6S20s (step 5) are resting in the large groove on each side of the axis.
10. Tighten the M6S20s in the BB (step 5) until the Y axis is firmly adhered to the BB, ensuring the X1P plate is allowed the full range of motion possible, and attach the X1P plate through the M5S50 screws used in wheel incorporation.
11. Place 2 M3Ns in the back groove of the MBXL200, bringing close to the centre.
12. Place 2 M3Ns in the front groove of the MBXL200, one at each end, leaving it lateral to the MBXL50.
13. Align a MakerBeam XL corner cube (MBXLCC) with each MBXL50 at the junction between the MBXL50 and the MBXL200 with the nuts aligned to the holes of the MBXLCC.
14. Affix each MBXLCC using these M3Ns and M3S6s to couple the MBXL50 to the MBXL200.
15. Gather 2 new MBXL50s and insert a M3N within the bottom groove of each. Align a MBXLCC with each M3N, and affix using a M3S6, ensuring alignment with the end of each MBXL50, forming a “L” shape.
16. Rest the unit from step 15 on the MBXL200 top surface, so that the angle created by the MBXLCC addition fits perfectly onto the corner of the MBXL200.
17. Insert a M3S6 into the build plate from below and attach a M3N to hold it loosely in place.
18. Slide a MakerBeam XL 150 mm (MBXL150) and a MakerBeam XL 100 mm (MBXL100) through the right and left M3Ns (step 17) of the X1P plate, respectively. Tighten the screws.
  a. NOTE: Ensure the shorter beam is on the same side as the Y axis motor to prevent obstruction of the full Y axis movement.
19. Insert 2 M3Ns into the top groove of each of the MBXL units from step 18, and 6 M3Ns into the top groove of the MBXL200, with 3 M3Ns on the left, and 3 M3Ns on the right.
20. Rest a MakerBeam XL T-bracket (MBXLTB) on the junction between the MBXL200 rod and the MBXL150 unit. Align the M3Ns with the holes in the MBXLTB.
21. Affix the MBXLTBs to the MBXL beams using M3S6s through each of the M3Ns.
22. Affix the MBXL50/MBXLCC units (step 13) to the back groove of the MBXL200 using two M3S6s, one for each of the M3Ns placed in the back groove of the MBXL200 (step 12).
  a. NOTE: These will be used to couple the XZ axes, so ensure they are in the correct position. To orientate these correctly, it may be necessary to perform steps 23 and 25 (not step 24) and mark the locations of the front groove of the Z axis on the MBXL200.
23. Place the 3D printed XYZ Coupler (3DXYZC) on the X1P plate through the inverted M5S50s used for wheel incorporation.
24. Affix the 3DXYZC unit to the plate using M5Ns (originally from X1P).
25. Insert the X1P Z axis bottom end into the front portion of the 3DXYZC, ensuring the Z axis stepper motor is resting on the back portion of the 3DXYZC.
  a. NOTE: You may need to loosen and then tighten the Z axis motor from the Z axis beam to align this properly.
26. Insert 2 M3 T-nuts (M3NTs) into each front groove of the Z axis beams.
  a. NOTE: This step cannot support the swapping of the nut with the screw, as the M3NTs have the required dimensions to support coupling.
27. Insert 2 M3Ns into the top groove of each of the MBXL50s (step 15).
28. Orientate a MBCB at the junction of the MBXL50 beams and the Z axis beams.
29. Loosely affix the MBCB to the MBXL50 beams using 4 M3S6s, one for each M3N.
  a. NOTE: Leave loose enough to be able to move the MBCB along the MBXL50 beams
30. Use 4 M3S12s to affix the MBCB to the Z axis beams through the M3NTs, ensuring the units rotate in a way to prevent their exit from the groove.
  a. NOTE: To do this, ensure the screw is in the threading of the M3NT, then use the screw to orientate the M3NT so that its long angle is perpendicular to the groove opening. Use the screw to pull the M3NT so that it is pressed against the groove, and then screw while maintaining the tension so the M3NT units maintain their orientation.
  b. NOTE: It may be necessary to establish the M3S12 with the M3NT through the MBCB **before** inserting the M3NT into the Z axis groove.
31. Tighten all screws from steps 29 until firmly attached while maintaining alignment. The X, Y, and Z axes are now fully coupled to each other in addition to the Y axis support rails.
  Optics Assembly (Fig. 5.)

**Fig. 5:**
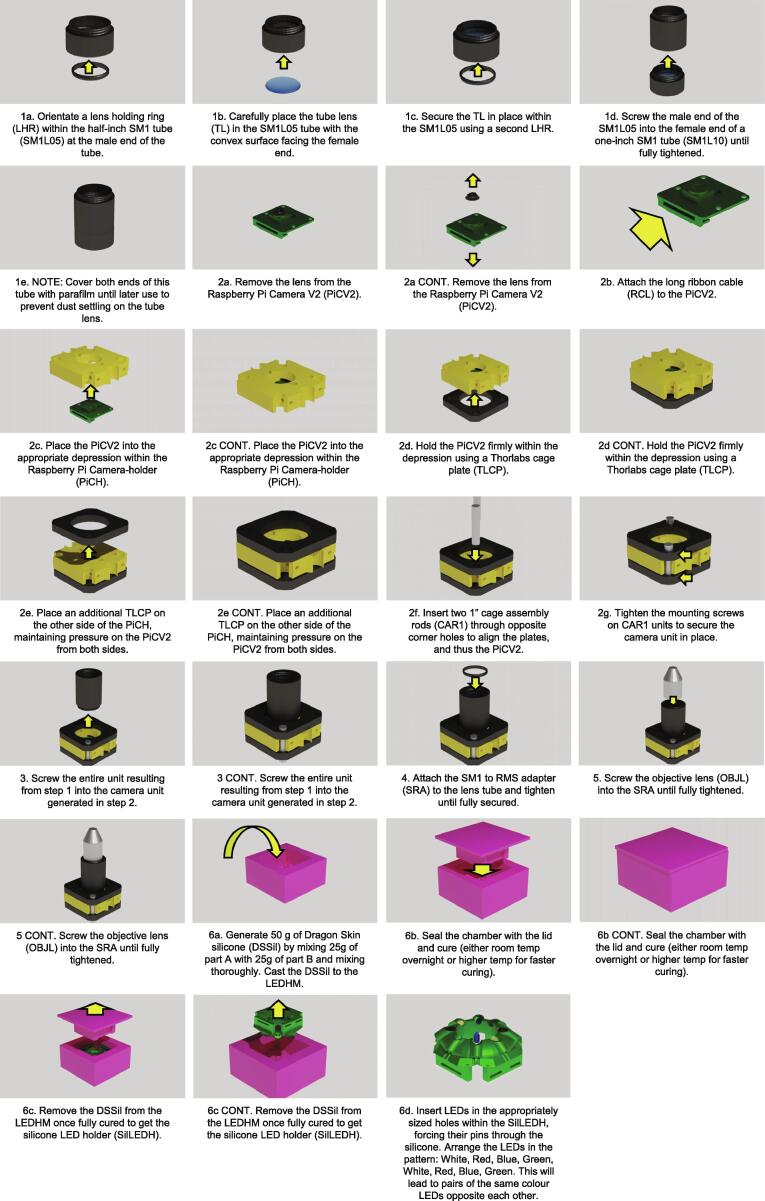
Incubot Assembly Visual Guide – Optics Assembly.
1. Construct the Optical Pathway
  a. Orientate a lens holding ring (LHR) within the half-inch SM1 tube (SM1L05) at the male end of the tube.
  b. Carefully place the tube lens (TL) in the SM1L05 tube with the convex surface facing the female end.
  c. Secure the TL in place within the SM1L05 using a second LHR.
  d. Screw the male end of the SM1L05 into the female end of a one-inch SM1 tube (SM1L10) until fully tightened.
  i NOTE: If fluorescent capabilities are required, incorporate the long-pass filter (LPF) into the SM1L10 using an LHR before attaching to the SM1L05
  e. NOTE: Cover both ends of this tube with parafilm until later use to prevent dust settling on the tube lens.
2. Construct the Camera Unit
  a. Remove the lens from the Raspberry Pi Camera V2 (PiCV2).
    i. NOTE: While it is possible to use pliers for this, several open source 3D printed options exist which reduce the risk of scratching the sensor and damaging the camera
  b. Attach the long ribbon cable (RCL) to the PiCV2.
  c. Place the PiCV2 into the appropriate depression within the Raspberry Pi Camera-holder (PiCH).
  d. Hold the PiCV2 firmly within the depression using a Thorlabs cage plate (TLCP).
  e. Place an additional TLCP on the other side of the PiCH, maintaining pressure on the PiCV2 from both sides.
  f. Insert two 1″ cage assembly rods (CAR1) through opposite corner holes to align the plates, and thus the PiCV2.
    i. NOTE: One rod should be to the left of the RCL when the PiCV2 is orientated with the lens facing upwards.
  g. Tighten the mounting screws on CAR1 units to secure the camera unit in place.
3. Screw the entire unit resulting from step 1 into the camera unit generated in step 2.
4. Attach the SM1 to RMS adapter (SRA) to the lens tube and tighten until fully secured.
5. Screw the objective lens (OBJL) into the SRA until fully tightened.
6. Construct the LED Holder
  a. Generate 50 g of Dragon Skin silicone (DSSil) by mixing 25 g of part A with 25 g of part B and mixing thoroughly. Cast the DSSil to the LEDHM.
    i. NOTE: It may be necessary to vacuum the silicone to remove large air bubbles, however this is unlikely to be necessary unless you are overly enthusiastic with your mixing.
  b. Seal the chamber with the lid and cure (either room temp overnight or higher temp for faster curing).
  c. Remove the DSSil from the LEDHM once fully cured to get the silicone LED holder (SilLEDH).
    i. NOTE: Use tools to try and pry free the lid portion from the mould. This is difficult and may break the lid of the LEDHM in the process.
  d. Insert LEDs in the appropriately sized holes within the SilLEDH, forcing their pins through the silicone. Arrange the LEDs in the pattern: White, Red, Green, Blue, White, Red, Green, Blue. This will lead to pairs of the same colour LEDs opposite each other (Fig. 6.).
    i. NOTE: Each LED should be orientated at 45° to the plane of the SilLEDH.

**Fig. 6:**
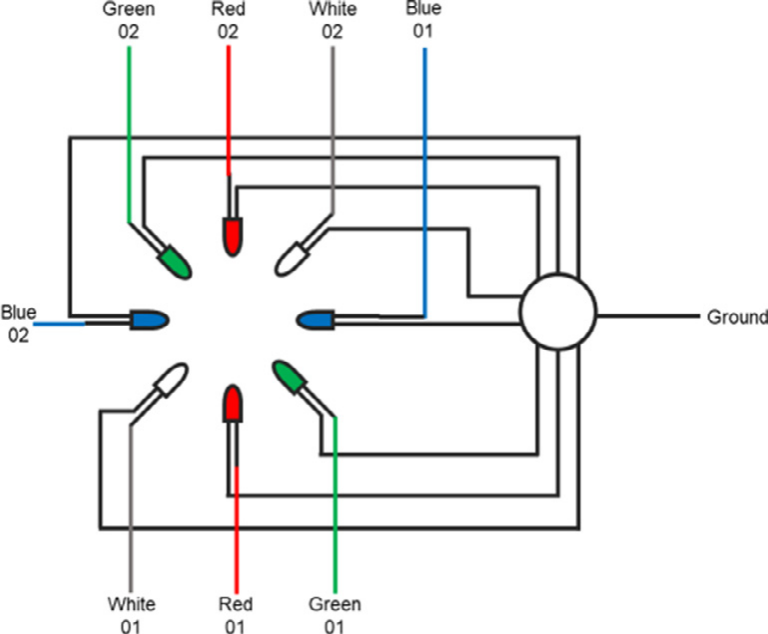
Incubot Assembly Visual Guide – LED Arrangement Schematic.
  e. Solder the LEDs to wires on their positive and negative pins.
  f. Collate the ends of the negative wires onto a single small metal ring and solder to secure.
  g. Solder a long wire to this metal ring, and then insert the free end into a female-female jumper wire.
  h. Label each of the positive wires appropriately.
    i. NOTE: The silicone moulded LED holder was easier to use, but harder to fabricate than a directly 3D printed holder. We have included the 3D model of the LED holder for users that prefer that option.

**Fig. 7:**
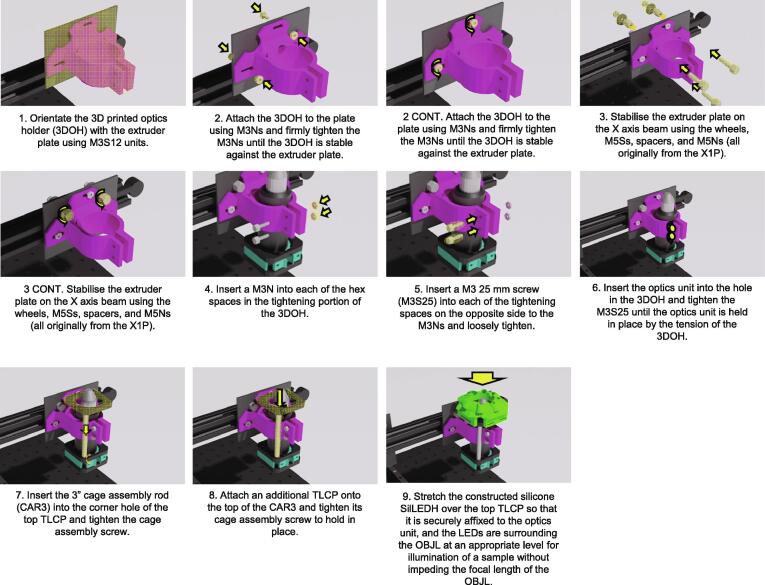
Incubot Assembly Visual Guide – Optics Coupling.

Optics Coupling (Fig. 7.)

1. Orientate the 3D printed optics holder (3DOH) with the extruder plate using M3S12 units.
2. Attach the 3DOH to the plate using M3Ns and firmly tighten the M3Ns until the 3DOH is stable against the extruder plate.
3. Stabilise the extruder plate on the X axis beam using the wheels, M5Ss, spacers, and M5Ns (all originally from the X1P).
4. Insert a M3N into each of the hex spaces in the tightening portion of the 3DOH.
5. Insert a M3 25 mm screw (M3S25) into each of the tightening spaces on the opposite side to the M3Ns and loosely tighten.
6. Insert the optics unit into the hole in the 3DOH and tighten the M3S25 until the optics unit is held in place by the tension of the 3DOH.
7. Insert the 3″ cage assembly rod (CAR3) into the corner hole of the top TLCP and tighten the cage assembly screw.
8. Attach an additional TLCP onto the top of the CAR3 and tighten its cage assembly screw to hold in place.
9. Stretch the constructed silicone SilLEDH over the top TLCP so that it is securely affixed to the optics unit, and the LEDs are surrounding the OBJL at an appropriate level for illumination of a sample without impeding the focal length of the OBJL.
  a. NOTE: Confirm a roughly 45° angle between the objective direction and the LED orientation to allow for oblique imaging later.

**Fig. 8:**
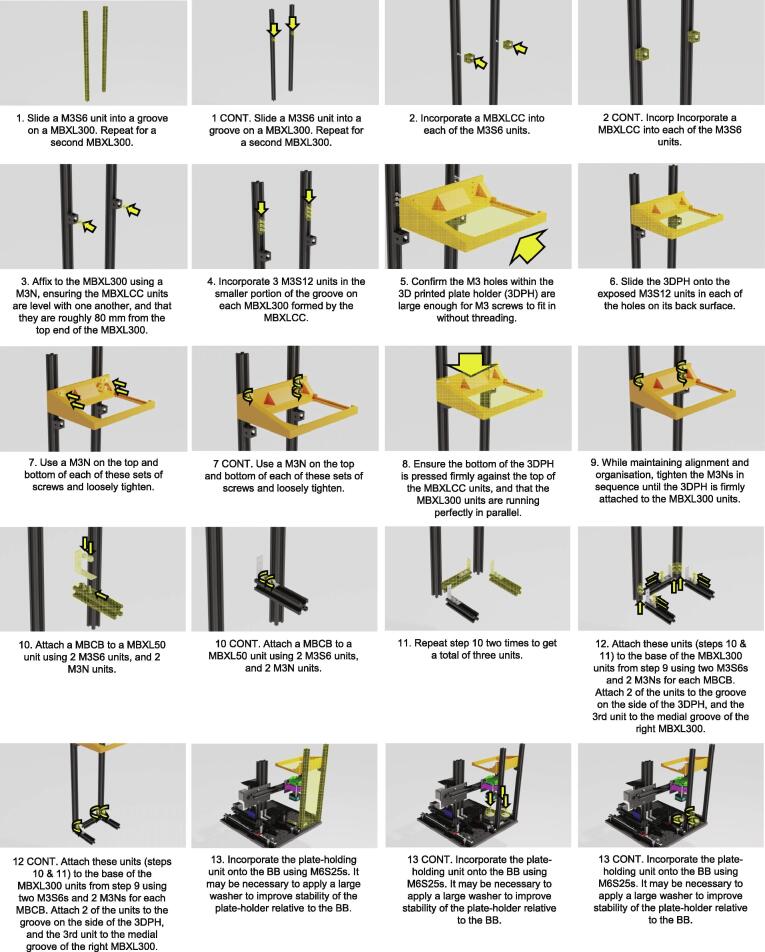
Incubot Assembly Visual Guide – Plate Holder Construction and Coupling.

Plate Holder Construction (Fig. 8.)

1. Slide a M3S6 unit into a groove on a MBXL300. Repeat for a second MBXL300.
2. Incorporate a MBXLCC into each of the M3S6 units.
3. Affix to the MBXL300 using a M3N, ensuring the MBXLCC units are level with one another, and that they are roughly 80 mm from the top end of the MBXL300.
4. Incorporate 3 M3S12 units in the smaller portion of the groove on each MBXL300 formed by the MBXLCC.
5. Confirm the M3 holes within the 3D printed plate holder (3DPH) are large enough for M3 screws to fit in without threading.
  a. NOTE: It may be necessary to use a Dremel or alternative to enlarge the holes, depending on the tolerances of your printer.
6. Slide the 3DPH onto the exposed M3S12 units in each of the holes on its back surface.
7. Use a M3N on the top and bottom of each of these sets of screws and loosely tighten.
8. Ensure the bottom of the 3DPH is pressed firmly against the top of the MBXLCC units, and that the MBXL300 units are running perfectly in parallel.
9. While maintaining alignment and organisation, tighten the M3Ns in sequence until the 3DPH is firmly attached to the MBXL300 units.
10. Attach a MBCB to a MBXL50 unit using 2 M3S6 units, and 2 M3N units.
11. Repeat step 10 two times to get a total of three units.
12. Attach these units (steps 10 & 11) to the base of the MBXL300 units from step 9 using two M3S6s and 2 M3Ns for each MBCB. Attach 2 of the units to the groove on the side of the 3DPH, and the 3rd unit to the medial groove of the right MBXL300.
13. Incorporate the plate-holding unit onto the BB using M6S25s. It may be necessary to apply a large washer to improve stability of the plate-holder relative to the BB.

**Fig. 9:**
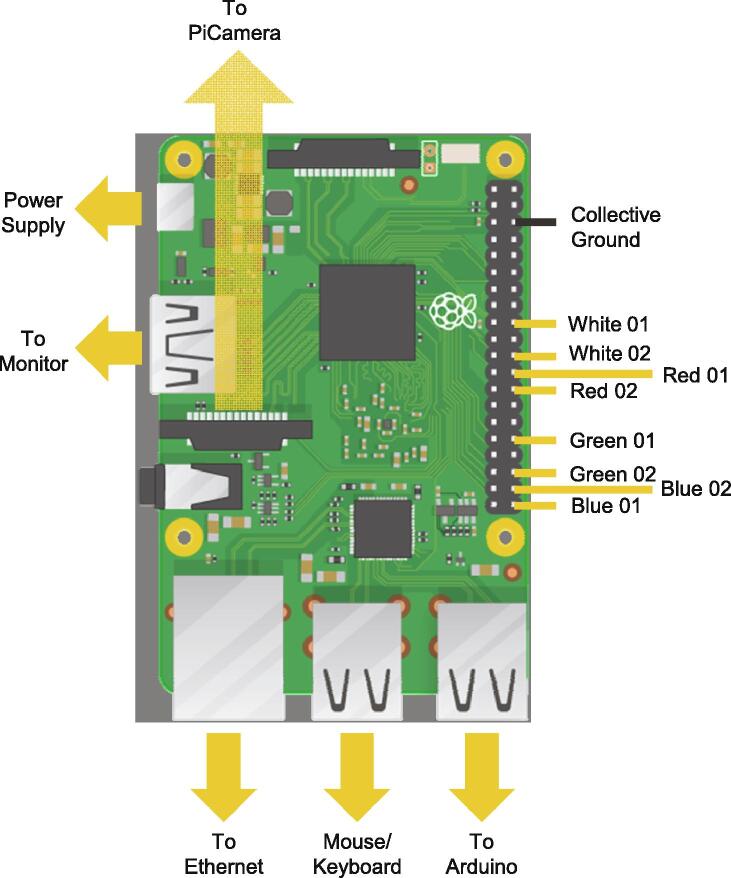
Layout schematic for Raspberry Pi. **Yellow arrows** indicate the location of cables to be attached to the Pi: power supply, monitor, ethernet cable, mouse/keyboard, and Arduino/CNC device. **Yellow lines** indicate path of wiring to from GPIO pinouts to LEDs (named). A single collective ground wire (**black line**) is used to ground all LED wires simultaneously. Raspberry Pi image from [4]. (For interpretation of the references to colour in this figure legend, the reader is referred to the web version of this article.)

NOTE: For a full guide on CNC motor shield establishment, please see [19].

Electronics (Fig. 9.)

1. Affix the heat sinks to the stepper motor drivers (SMD) [20].
2. Apply any connectors to the microstepping pins on the CNC motor shield (CNCMS) that you wish, keeping note of the impact this will have on microstepping and torque ability.
3. Insert the SMDs into the appropriate positions for the X, Y, and Z axes of the CNCMS, leaving the A axis without a driver and ensuring the SMDs are orientated correctly.
4. Attach a red wire to the positive inlet of the CNCMS, and a black wire to the negative inlet of the CNCMS.
5. Solder/connect these wires to the corresponding wires/connectors of a 12 V/1250 mA power supply.
6. Connect the stepper motor cables to their appropriate pins on the CNCMS: X-X, Y-Y, Z-Z, leaving the A pins blank.
  a. NOTE: Depending on the distance from your motors to your CNC motor shield, it is likely necessary to replace the cables that come standard with the X1P with a longer alternative.
7. Attach the CNCMS to the Arduino Uno R3 (AUR3), ensuring all pins are firmly in their appropriate openings, taking care not to bend or damage any pins.
8. Connect the AUR3 to the Pi3B + using a USB cable.
9. Establish the GPIO pins and any additional cables as specified (Fig. 9.).
10. Connect the AUR3 to the Pi3B + using the USB cable.

Incubator Incorporation

1. Detach any cables connecting the *Incubot* to the Pi3B+, AUR3, or power supply. Leave the connections in place on the *Incubot* for ease of re-connecting.
2. Ensure a grating is present on the bottom rung of your incubator and confirm this is securely in place and level.
3. Remove enough gratings above this to facilitate the maximum height of the *Incubot* (Z-axis lead screw).
4. Spray down the disconnected *Incubot* with 70% ethanol and use an ethanol-soaked wipe to scrub the build as much as possible.
5. Apply WD40 or alternative lubricant to all bearings, moving parts, and parts potentially prone to corrosion to prevent damage from the humidity within the incubator.
6. Re-spray the *Incubotwith 70% ethanol*, and place quickly onto the bottom grating of the incubator. Ensure the *Incubot* is placed with the Y axis motor towards the hinge of the incubator, to allow for the full range of motion in X, Y, and Z axes.
7. Ensure all wires and cables connected to the *Incubot* are led safely out of the incubator towards the opening side of the incubator. Orientate the X, Y and Z axes so that the Pi Camera is at the furthest possible distance from the opening of the incubator
8. Leaving a little bit of slack in the cables, affix them to the external incubator wall using electrical tape or an alternative adhesive, making sure to spread the cables so that there are no major gaps in the incubator seals that could allow for gas leakage while the doors are closed.
9. Re-connect the cables to their appropriate positions in the Pi3B + and the AUR3.
10. Return the X, Y, and Z axes to their “resting position”. For our lab, we choose to bring the Y axis as far away from the opening as possible, and the X axis as close to the opening as possible. The Z axis will be left at a height suitable for future imaging, however the level of the Z axis is not important at this point in the construction.

Software Installation and Setup

1. Install Raspbian onto Raspberry Pi (via Noobs is recommended)
2. Install dependencies (using Python 3.7.3):
  a. Kivy
    i. NOTE: For a full description on getting Kivy to work on your Raspberry pi, please follow a detailed tutorial such as on the Kivy website [21]
  b. NumPy
  c. PIL
3. Install Additional Software
  a. Arduino IDE
  b. GRBL (install directly onto the Arduino, this will allow for cartesian control of the XYZ axes)
  c. VNC (or alternative remote screen system compatible with Raspbian)
4. Download all necessary files from the online repository (https://doi.org/10.17605/OSF.IO/ES3HR) onto the Desktop

## Operation instructions

### Initial Operation

Once the hardware has been established, code imported onto the Pi3B+, and the software is installed correctly, the device is ready to be calibrated. The depth of calibration will depend on the intended use of the *Incubot* and can be updated at any time. Calibration focuses on the key steps:

1) Validation of Motion
2) Calibration of Plate and Well Location
3) Objective Lens Calibration
4) Imaging Folder Generation

### Validation of Motion Settings

This section will cover the details of validation of *Incubot* motion prior to starting any experiments, describing how the user should confirm that the *Incubot* was capable of following movement commands reliably. The user should assess how closely the motion we requested is followed by giving the *Incubot* commands to move a specified distance in a specified direction while the optics is focussed on a scale graticule. Using this graticule, we can then calculate the actual distance moved by the *Incubot* and compare this to the commanded motion. G-code is a method of communicating specific instructions to a 3D printer to command it to move in a specific direction at a specific velocity. In the case of Arduino hardware, GRBL then converts the command into stepper motor function, rotating the motor for each axis in such a way that the hot end of the printer will end up in the correct final location. The conversion of the distance commanded to a specific number of stepper motor rotations can be adjusted within the Arduino IDE using GRBL.

To start, calculate the theoretically expected number of steps the stepper motor will need to rotate to move one mm based on the stepper motor brand (e.g. Nema-17) and the amount of microstepping used. Open the Arduino IDE and open a serial monitor. Type “$$” and press the “enter” button to bring up the current settings. Alter your specific setting by typing “$” and the number designation of the setting, followed by an “=” sign, and the new setting value. Then, open the source code for the script “MotionValidationGUI.py”. Make sure a folder is correctly specified in line 73, for your images to be saved to. Then, run the script from your Raspberry Pi terminal. This will open a GUI that will guide you through motion validation. Use the motion control system on the left side of the screen until the scale graticule is located centrally within your FOV. Once you are ready, select the “Begin Imaging” button. This will send G-code commands to the *Incubot*, imaging prior to the command and then imaging again after the command. It will save these images to the pre-defined folder in the source code. Once imaging is complete, there should be 800 images saved within the folder, each labelled according to the axis, direction, repeat number, and whether it is the start image or the end image. Transfer all image files from the appropriate folder to a standard computer/laptop with FIJI [22] installed. Open ImageJ and ensure the program is up to date, updating the program if necessary. Open the script “PairwiseStitchingMotionValidation.ijm” within ImageJ. Alter the “folder” parameter to reflect the folder you have placed your images into. The ImageJ script will sequentially stitch images within this folder, two at a time, recording the pixel translation along with other details of the stitching process. If a results box is currently open, ensure you clear this, then run the stitching script and leave to complete. Once the script has completed, you will be left with a results box with 1600 lines of information.

Copy and paste this information into the first column of the sheet “Raw Data Input” of the template excel file “Movement Validation Template.xlsx”. The excel sheet will automatically extract the important information from the cells and bring the data into the subsequent sheets. Use an image of your scale graticule taken by the *Incubot* and determine the pixel-to-µm conversion factor (we recommend using ImageJ). Input the number of pixels per µm into cell C1 within the following sheet “Data Visualisation. This will automatically convert your raw pixel data into real distances in µm. The tables should now update, informing you of the average disparity between commanded motion and observed motion. If the median observed motion is more than 5% greater or smaller than the commanded motion, then calculate the over-movement or under-movement. If movements are unexpectedly inaccurate for a specific movement range, attempt to stitch a selection of images for this movement range and observe the result; it may just be a stitching issue. If it is an actual movement issue, adjust the parameters within GRBL. Repeat the validation process through the GUI and determine if the change was effective.

### Calibration of Plate and Well Location

**Fig. 10:**
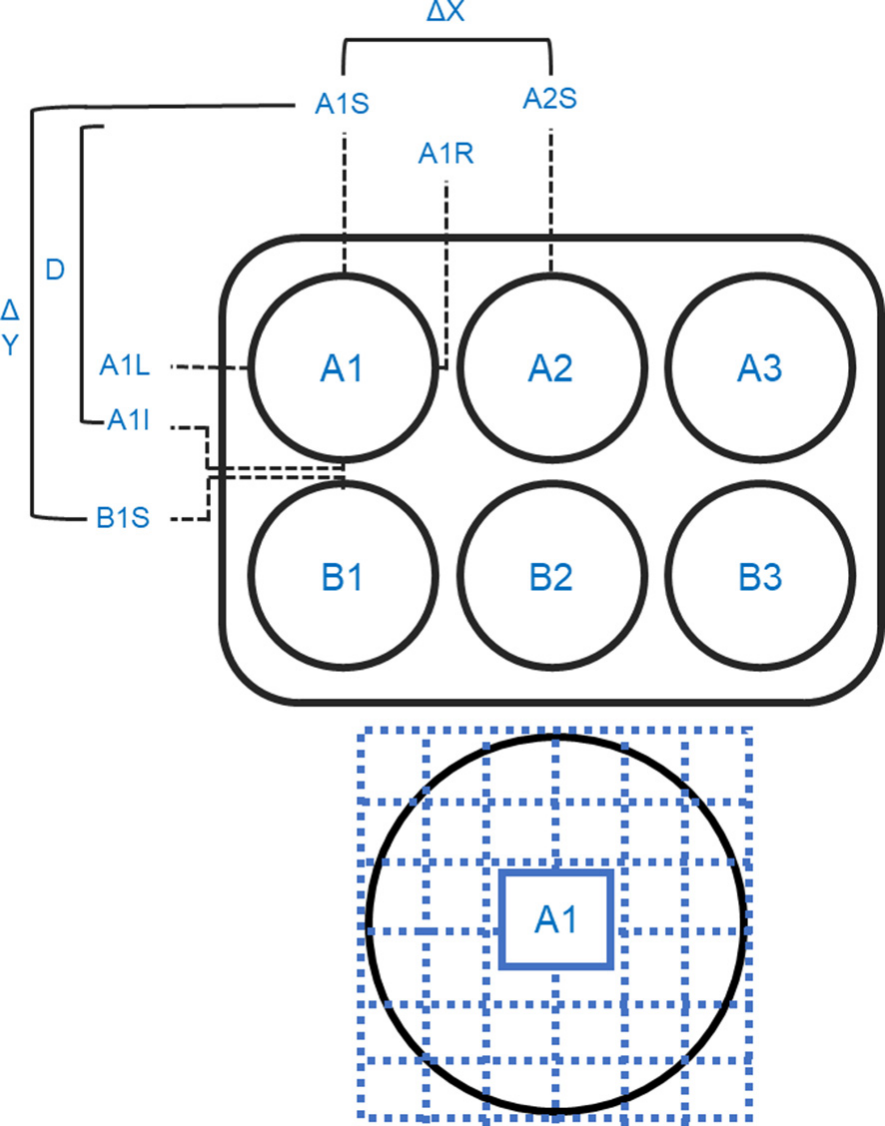
Schematic Representation of a 6-Well Plate with Landmarks for Well Location Annotated. The positional points for well A1 are defined as the top-most (A1S) the bottom-most (A1I), the left-most (A1L) and the right-most (A1R) points on the well. The difference between A1S and A1I will give the diameter of the well (D), as will the difference between A1L and A1R which should be the same. The difference between the same landmark on adjacent wells will give the distance in X (A1L-A2L or A1R-A2R) between wells (ΔX), and the distance in Y (A1S-B1S or A1I-B1I) between wells (ΔY). Full available imaging area is shown below, with segments for later user selection highlighted.

For the *Incubot* to accurately image a plate, it needs to be informed of where your plate is relative to its home position. To do this, place a plate of the type you wish to calibrate in the plate holder. Run the python script “WellLocationGUI.py” and wait for the camera to initialise. Select the type of plate you are calibrating for, then use the X, Y, and Z motion control system on the left side of the screen to orientate your plate into the FOV. For the *Incubot* to calculate well locations, you need to define key well landmarks as outlined in Fig. 10. Use the motion control system to orientate each key well landmark into the centre of the FOV and then press the corresponding button for that landmark. Once you have selected all the key landmarks, select the “Save Parameters” button. This will determine the starting location for this plate type and will calculate the necessary spacing between individual wells. It will output five arrays into the terminal. Simply copy these arrays into the *Incubot* GUI python source code into the corresponding section for your well type. Once performed, provided the plate location is stable between experiments, this calibration will only need to be performed once for each plate type. If at any point the plate-holder design is rearranged, or altered in any way, this calibration step will need to be re-performed.

### Objective Calibration

If using a different objective lens to that described in this build, you will need to adjust the objective lens parameters within the “IncubotGUI.py” script (lines 682–708). Simply attach the objective lens to be used (infinity conjugate is necessary to keep the light path short enough for use, however a modified longer lens tube would facilitate use of a finite conjugate objective lens), and open the script titled “CameraPreview.py”. Adjust the camera parameters to capture and display a preview of the appropriate dimensions for normal *Incubot* functioning, save the script, and run the script using Python3 via the terminal. Establish focus on a graticule with known dimensions and exit out of the script. Un-comment lines 41–43 (“CameraPreview.py”) to enable image acquisition, input desired image save location (line 41) and image name (line 42), save the script, and re-run from the terminal. Transfer the resultant image to a desktop computer and determine the scale factor (pixels per µm) for the objective lens using FIJI. Use this scale factor to determine the size of the FOV in mm (both X and Y). Multiply these numbers by a factor of 0.8 to give the X and Y increments when using this objective to ensure enough image overlap for image stitching during image processing and analysis. Update the “IncubotGUI.py” script at line 682–708 within the magnification setting functions with the modified values for xMod and yMod, depending on the magnification you are calibrating for. If using a different objective lens magnification, it would be worth updating the label for one of the provided magnification buttons, along with updating the function to reflect your different objective lens specifications.

### Image Folder Generation

Additional parameters at the start of the “IncubotGUI.py” script specify folders of importance for image saving. It is crucial that you determine and create an appropriate imaging folder for centralised image saving, whether that is on the raspberry pi itself or on an external storage device. Create a folder in your desired location and paste the folder location into the variable “folder” (line 105, “IncubotGUI.py”). If you possess a subscription to a large-volume Dropbox, I would recommend following the instructions within [23] and establish the settings as desired. Rename the Dropbox Upload script “dbupload.py” and uncomment the import bpupload (“IncubotGUI.py”, line 22), and ensure it is in the same directory as “IncubotGUI.py”. Please note that the *Incubot* will upload between imaging sweeps and will wait for upload to complete prior to beginning the next sweep, potentially impacting time between sweeps. If Dropbox upload is selected, ensure your upload speed is sufficient to transfer all image files from an imaging sweep in the time between sweeps.

### Routine Operation

**Fig. 11:**
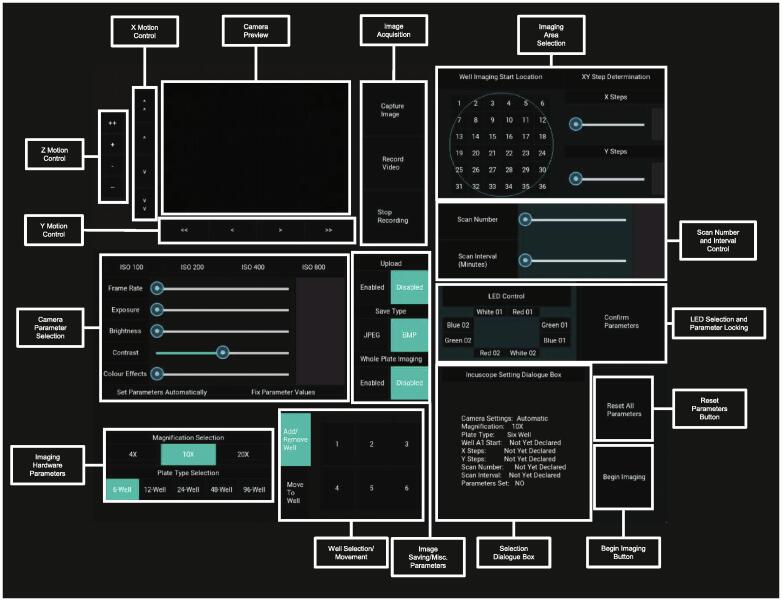
Incubot GUI with Key Areas for User Selection Annotated.

**Fig. 12:**
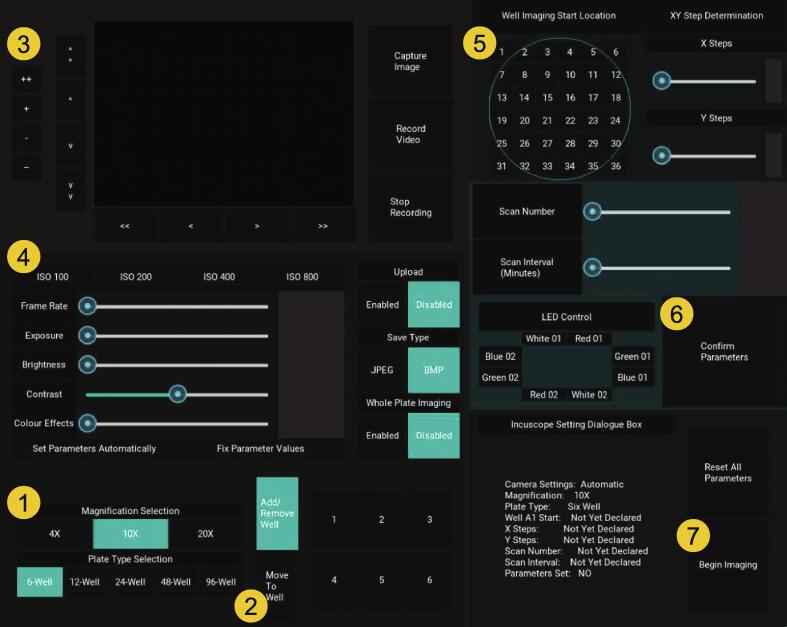
Quick-Start Operational Guide to Routine Use of the Incubot GUI. 1) Select your hardware settings (magnification and plate type). 2) Select the “Move to Well” button and select a well of your choice from the grid layout to the right of it. 3) Adjust the X and Y positions until cells are in view and adjust the Z position until cells are in focus. 4) Adjust the image acquisition parameters if needed and alter any image acquisition parameters you wish to. 5) Select an appropriate start location for your experiment and select the number of images in the X and Y axes you wish to collect. Alter the scan number and scan interval parameters to suit your experimental needs. 6) Once all settings are to your liking, confirm the parameters by pressing the “Confirm Parameters” button. 7) Once sure that imaging is established to your liking, press the “Begin Imaging” button to begin the process of imaging. The Incubot will be unresponsive during the imaging run, so if emergency stopping is required, the Pi must be physically switched off and back on again. Note that this will result in the loss of positional XYZ information and the Incubot will assume the position it is left in is X0Y0Z0. You may need to manually move the Incubot to its X0Y0, or via the Arduino IDE once rebooted using negative X and Y coordinates.

An annotated version of the *Incubot* GUI is provided in Fig. 11. For a quick-start GUI guide, please review Fig. 12. A video of the *Incubot* during movement has also been uploaded to the OSF repository (https://doi.org/10.17605/OSF.IO/ES3HR).

You may manually determine the appropriate Z level for general imaging by using the “CameraPreview.py” script with a small image preview in conjunction to sending G-code directly using the Arduino IDE program. To do this, establish an image preview and send G-code to move the X and Y axes to an appropriate coordinate for sample visualization. Adjust the Z axis until focus is achieved. Command this location as the new Z0 point using the G-code command “G92Z0”. Alternatively, this can be performed within the *Incubot* GUI. Select your objective lens magnification and plate-type (bottom left corner). The box to the right of this section will update to reflect the plate type you have selected. Press the “Move to Well” button to use the simulated plate to move to the default XY coordinate specified for the selected well, which will not have the well base visible. Use the X and Y motion control sections to move the FOV to a region within the well where cells should be visible. Use the Z motion control section to adjust Z axis until focus is achieved. It may be necessary to adjust your Camera Parameter Selection options to optimise image preview/acquisition parameters. The LED “White 02” will be active by default, so you may also need to select alternative LED(s) using the LED Control Box (middle right). Select/deselect options relating to Dropbox uploading, image file save type, and whole-plate imaging using the buttons within the Image Saving/Misc. Parameters section. If you would like to acquire an image or recording now for your records, you can do so using the Image Acquisition section to the right of the Camera Preview. If you wish to exclude certain wells from imaging, press the Add/Remove Well button and then select the wells you wish to exclude (excluded wells will be highlighted in dark blue).

To alter parameters relating to your imaging experiment needs, use the right hand side of the GUI. The imaging area by default will start outside of each well, at the minimum X and Y coordinate location as defined by the user during plate and well calibration. To alter this start location, select an appropriate button within the Well Imaging Start Location layout. A representative well is shown using a blue circle to allow for visualization of your rough start location. Select the area you would like to image using the XY Step Determination section to select your X and Y imaging dimensions in terms of number of images along the X axis and images along the Y axis. Below this, select the number of times you would like to scan the plate, along with your desired interval between scans (start time to start time). It is possible to set impossible requirements here by requesting an interval that is shorter than the actual time required for a single scan. Under these conditions, the *Incubot* will begin scans immediately after the previous one, however it will not inform you if this is expected to be the case. You can reduce the time required for a complete scan by reducing the area of imaging, or by saving in JPEG format as opposed to BMP. Ensure the LED(s) you would like to use during imaging are selected in the LED Control section (selected LEDs will be highlighted in blue). Once you are happy with your parameter selection, set the parameters using the Confirm Parameters button.

At this point you may wish to review your settings selection by observing the *Incubot* Setting Dialogue Box. If you wish to change parameters you may either alter the slider/button individually or press the Reset All Parameters button to begin the process from scratch (this will only affect parameters, and not the current *Incubot* coordinates). Once all parameters are to your liking, you are ready to begin imaging by pressing the Begin Imaging button. This will trigger the start of the scanning process. The *Incubot* will begin imaging at the first non-excluded well in the order of A1, A2, A3… B1, B2…etc. Once imaging has concluded, provided you have opted not to automatically upload your files to Dropbox, exit out of the GUI by pressing the Esc button on your keyboard. The imaging folder will be within your selected directory ready for transfer to a desktop computer for image processing, stitching, and analysis.

### Routine Maintenance

Due to the use of the *Incubot* within a tissue culture incubator, regular coating of parts with WD-40 (or alternative lubricant) is required to prevent corrosion of parts. We recommend performing this every 2 months, however more frequently is not problematic.

Cleaning should be carried out when needed by removing the *Incubot* from the incubator and scrubbing down the build using tissue soaked in ethanol. WD-40 should be reapplied following each cleaning. Provided there are no tissue culture infections or visible build-up of matter, cleaning of the build can be kept to roughly every 6 months.

## Validation and characterization

### Hardware Validation

#### Plate Stability

**Fig. 13:**
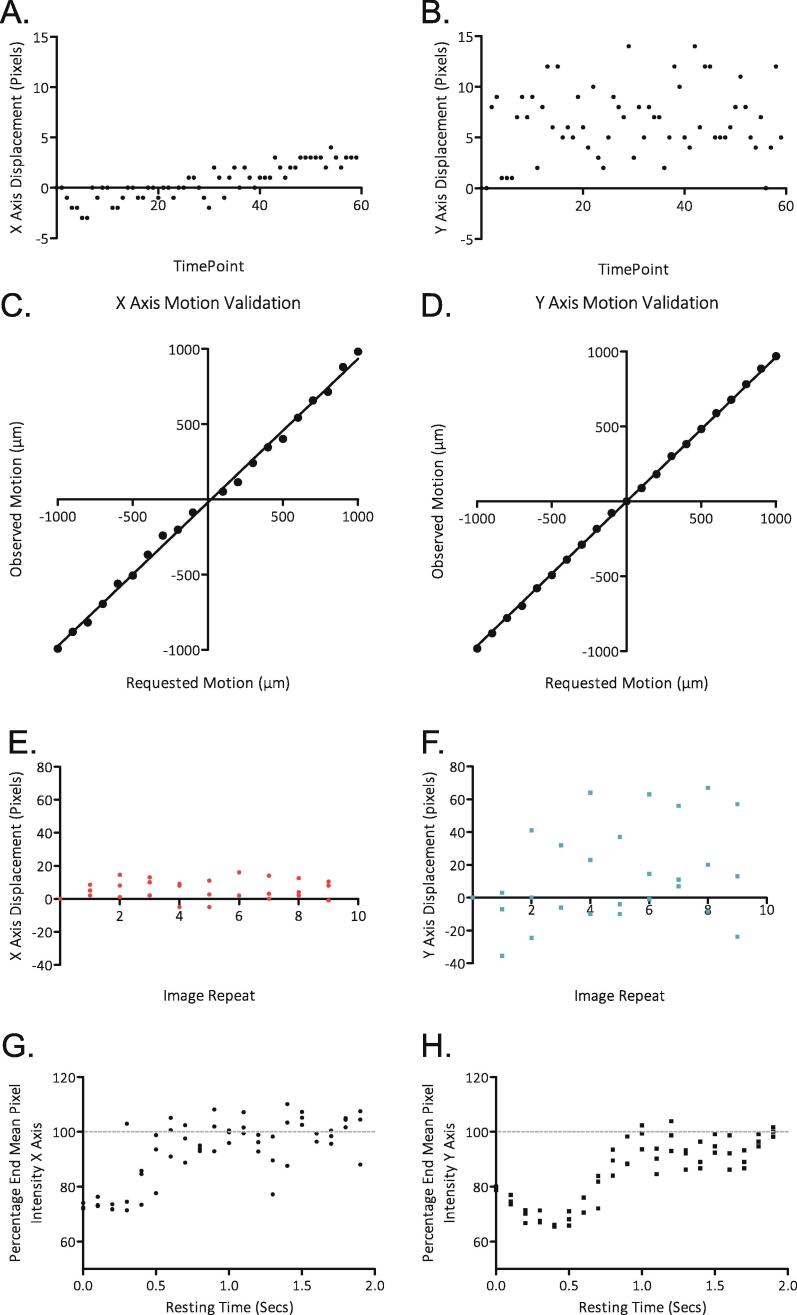
Stability and Motion Validation of the Incubot. A) Deviation of an X coordinate of a landmark within an image over 60 min of imaging. B) Deviation of the Y coordinate of a landmark within an image sequence over 60 min of imaging. C) Distance moved by the Incubot in the X axis compared to requested distance via G-Code. D) Distance moved by the Incubot in the Y axis compared to requested distance via G-Code. E) Deviation in X coordinate location of a landmark when the Incubot is commanded to move from a set location from a different location. F) Deviation in Y coordinate location of a landmark when the Incubot is commanded to move from a set location from a different location. G) Sharpness [7] of images taken by the Incubot following movement in the X axis with variable rest time delay durations. H) Sharpness [7] of images taken by the Incubot following movement in the Y axis with variable rest time delay durations.

Plate stability was assessed by imaging a single location of a USAF 1951 resolution testing slide every minute for 60 min (“StationaryStabilityTesting.py”). The coordinate location of a defined high-contrast line corner within the image was manually determined, and the displacement over time was calculated in both the X and Y axes. The X axis (Fig. 13A.) showed a higher level of stability than the Y axis (Fig. 13B.), and only moved a on average +/- 1.3 µm over the time span. The Y axis showed greater displacement (+/- 5.8 µm), indicating a minor degree of motion between images, however as the variations showed no trend over time (linear regression, P = 0.1998), there is no evidence of persistent drift.

### Movement Fidelity

Movement fidelity was calculated according to the protocol established earlier in this manuscript (Operation Instructions > Validation of Motion Settings). Motion was requested in the range of 0.1–1.0 mm (Y axis) or 0.1–1.0 mm (X axis) in 0.1 mm increments. Following optimisation of the GRBL parameters controlling steps per mm, motion was highly reliable for a given requested motion, showing very little variability (<5% variation from requested motion) (Fig. 13C, D.).

The reliability of coordinate motion was also calculated by repeated imaging of a fixed tissue culture plate. The *Incubot* was commanded to scan single points within wells 10 times, moving back to the home location between each imaging sweep. Location of a structurally defined point (the termination of a cell process) was manually determined and the displacement was compared between imaging sweeps for 3 wells in both X and Y axes (Fig. 13E, F.). Y axis repeatability showed more variation than the X axis. This is likely due to greater instability caused by the Y axis carrying the mass of both the X and Z axes, compared to the X axis just carrying the optical components. This increased Y axis displacement is not significant enough to disrupt normal image processing/stitching procedures.

### Minimum Time Between Movement and Imaging

As the *Incubot* hardware rests within a tissue culture incubator, it is impossible to completely protect against vibration and its effect on image quality. The major source of vibration within the incubator comes from the *Incubot* itself during periods of X or Y axis movement, necessitating a rest period between movement and image acquisition. To calculate the minimum delay required between movement and imaging, testing was performed to identify the shortest necessary period between movement and imaging. The *Incubot* was set up focused on human dermal fibroblasts (HDF) fixed with 4% paraformaldehyde. We used a script (“MotionRestTesting.py”) to trigger movement of the optics 1 mm in either the X or Y axis and collected images at 0.1 s intervals over a period of 2 s. Stability was measured by the sharpness of the image, calculated using the ImageJ plugin “Microscope Focus Plugin” [24]. Stability was achieved 0.6 s after movement command in the X axis, and 1.0 s after movement command in the Y axis (Fig. 13G, H.).

### Resolution Testing

**Fig. 14:**
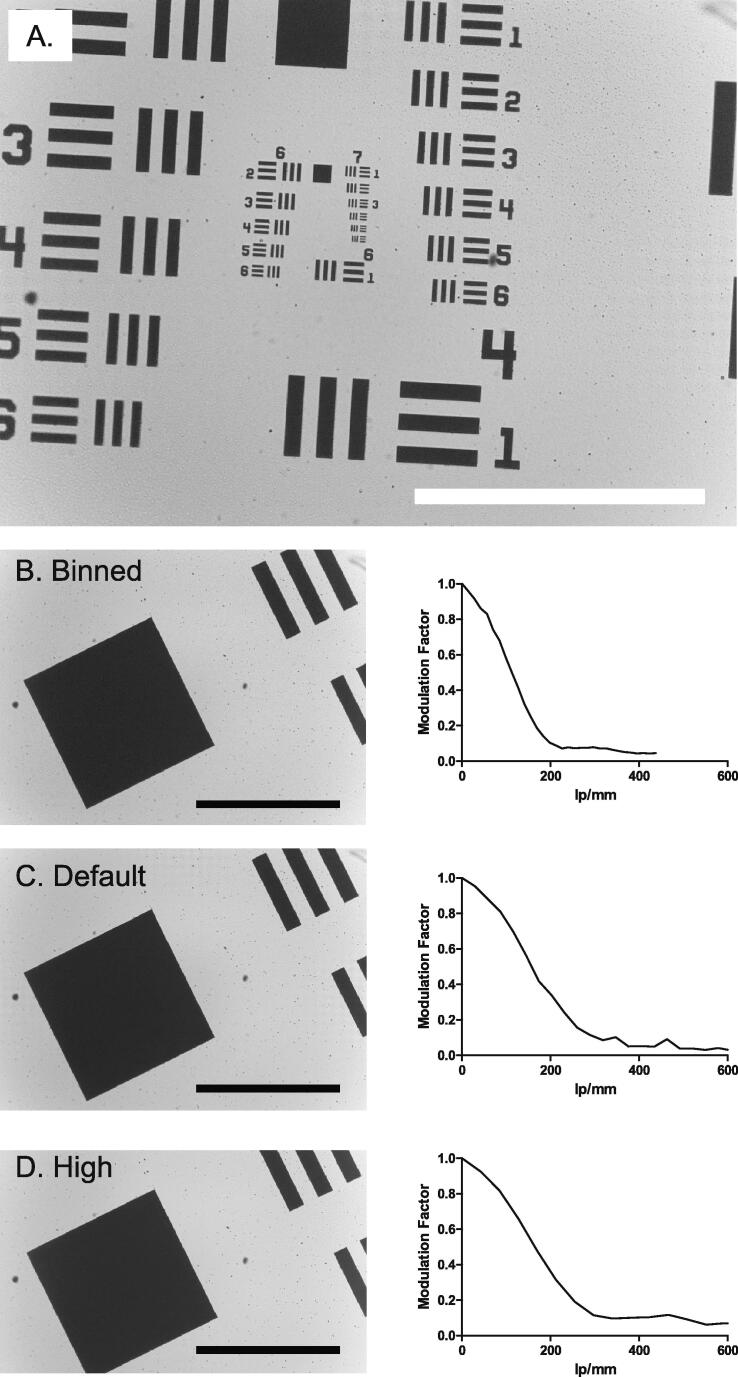
Optical Validation of the Incubot**.** A) USAF 1951 resolution testing grid representative image taken on Incubot at 10X magnification using collimated white light transmission illumination. Analysis of resolution using slant edge MTF with comparisons between a highly binned image (820x616 pixels) (B), the pixel resolution used for Incubot function (1680x1200 pixels) (C), and a higher pixel resolution (2464x1500 pixels) (D). Slant edge MTF [9] was calculated from the major right-hand slope present in each of these images, and is shown next to each tested image. Modulation factor is labelled on the y-axes, while spatial resolution measured in line pairs per millimetre (lp/mm) is labelled on the x-axis. Scale bars represent 500 µm.

Maximum image resolution was determined by imaging a USAF 1951 resolving power grid (Fig. 14A.) [25] and also by calculating the slant edge modulation transfer function (MTF) in FIJI [26]. The FOV was split into 20 subregions in a 4x5 grid, and the test pattern was imaged (using transmitted white light illumination) with the smallest resolvable elements moved between each of the subregions. The smallest lines clearly visible in each quadrant was recorded and converted into its equivalent lines.mm^−1^. The maximum testable resolution able using our USAF 1951 slide was a series of lines with a density of 228.1 lines.mm-1 (group 7 element 6). This structure was resolved within all subregions of the Incubot FOV, meaning that resolution for all subregions is at least 228.1 lines.mm^−1^. Slant edge modulation transfer function (MTF) was also assessed on an image of the USAF 1951 slide taken by the Incubot using transmission illumination at three different pixel resolutions: high resolution (2464x1500), the default Incubot resolution (1680x1200), and a 4X binned resolution (820x616). The MTF curves are presented in Fig. 14B, C,D. Increases in resolving ability was observed in the *Incubot* settings compared to the binned settings, while increasing pixel resolution further did not result in similar increases in resolving ability. Therefore, the *Incubot* pixel resolution settings were applied to balance resolving ability with saved image storage space required and time required for image saving. Users should note that depending on their own requirements, the binning automatically used within the *Incubot* may result in under-sampling of their images. We encourage users to adjust these parameters based on their own requirements.

### Validation of imaging with multiple light sources

#### Reflected and Oblique Illumination

**Fig. 15:**
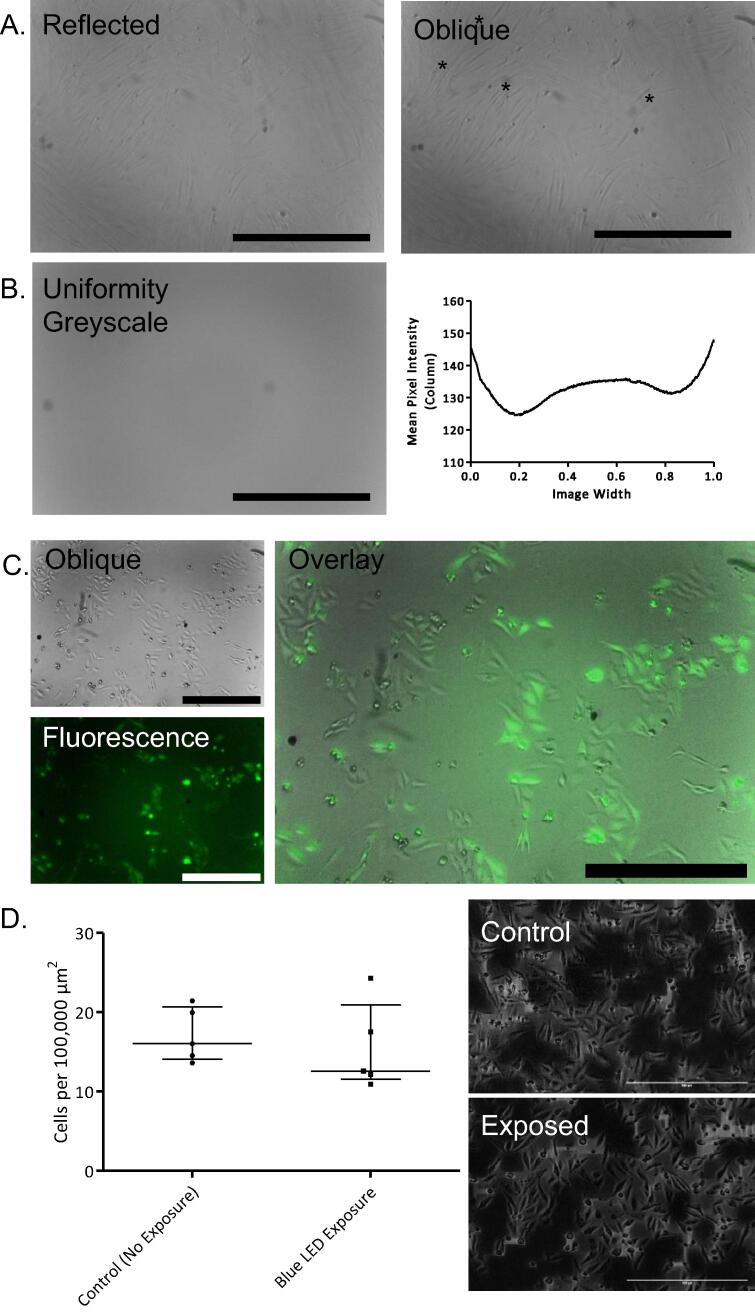
Image Acquisition Validation of the Incubot. A) Representative image of live HDF cells using dual white LED illumination (reflected) or single white LED illumination (oblique) with processes uniquely identified using oblique illumination highlighted with (*) (scale bars = 500 µm). B) Uniformity of fluorescent imaging assessed using a Thorlabs green fluorescent slide (FSK2) in greyscale (scale bar = 400 µm) with mean pixel intensity value from each column along the image’s width. C) Live HeLa-GFP visualization using oblique illumination, blue LED fluorescent illumination (acquisition exposure time < 1 sec), and overlay (scale bars = 500 µm). D) Assessment of phototoxicity revealed no significant difference in cell density between LED-exposed and control cell after 24 h of repeat illumination of live HeLa-GFP cells (scale bars = 500 µm, representative images subjected to background subtraction processing in ImageJ). (For interpretation of the references to colour in this figure legend, the reader is referred to the web version of this article.)

HDF cells were cultured in a 6-well plate and imaged using both white LEDs, then just a single white LED to compare reflected white illumination with oblique white illumination. Oblique illumination revealed additional cellular details not visible with dual white LED illumination, highlighting the utility of low-cost single LED illumination at a 45° angle for visualising cellular morphology (Fig. 15A.). Introducing excitation light at an oblique angle eliminates the need for dichroic mirrors along the optical path, but it does introduce issues relating to illumination uniformity. The LEDs used in our build were dome-shaped, and the blue, red, and green LEDs emitted light in a 70-80° arc from their dome. The white LEDs emitted light in only a 55° arc. In our work, the unevenness of illumination in reflected/oblique imaging has not caused any major problems, and grid/collection stitching using ImageJ is still capable of stitching adjacent FOVs correctly.

### Fluorescence

Fluorescence illumination uniformity was assessed by illuminating a fluorescent slide (Thorlabs, FSK2, green emission) using both blue LEDs (Fig. 15B.). While the slide was illuminated to the point of fluorescence, the illumination was not completely even. For our purposes, the uniformity is sufficient, however other users may wish to improve the illumination uniformity if performing quantitative fluorescent analyses. For fluorescence imaging the *Incubot* needs to be capable of fluorescence illumination and detection, but importantly because this system needs to be capable of fluorescence imaging repeatedly over a long time frame, it should not induce significant photodamage to cells over the course of this imaging. We assessed this by culturing commercially available HeLa cells stably expressing GFP [27]. GFP was excited by both blue LEDs and the emission was detected and overlaid well with oblique images taken in the same coordinate location immediately after (Fig. 15C.). To assess phototoxicity induced by repeated imaging, two 6-well plates of HeLa cells were seeded at a density of 15,000 cells per well and left for 4 h to allow for cell attachment. One plate was covered from light within the incubator, while the other was subjected to repeated imaging under blue LED excitation every hour for 24 h. Following the imaging protocol, the light-protected plate was imaged once in the same relative locations as the imaged plate. Cell density was calculated at this 24-hour point for all wells exposed to the LEDs (fluorescent) and all wells protected from light (negative control) (Fig. 15D.). There was no significant difference between the density of cells in wells exposed to the blue LEDs compared to control wells (Mann Whitney, P = 0.4206), indicating no significant impact of LED exposure on cell viability.

### Imaging area

**Fig. 16:**
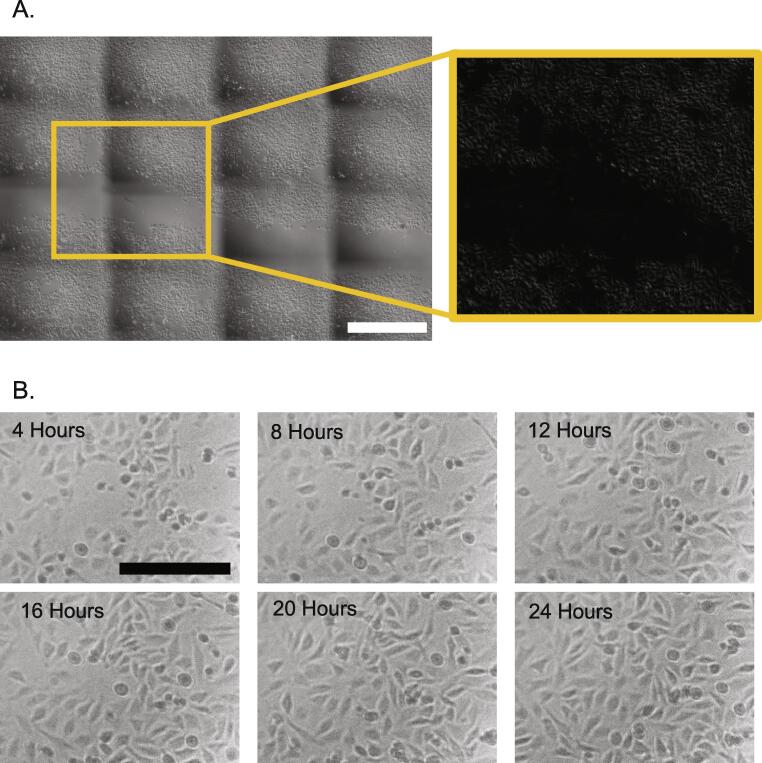
**Validation of Image Acquisition.** A) Incubot was established using automated acquisition settings and requested to perform a 7 × 6 FOV acquisition of a HeLa-GFP plated tissue culture well 17 h post-seeding for a preliminary scratch assay, cropped to roughly 4 × 4 FOVs. Acquired images were stitched in ImageJ using “Grid/Collection Stitching” plugin, resulting in full image shown. Scale bar indicates 1000 µm. A section of the image was then subjected to background subtraction via ImageJ, to show how this processing can remove the impact of uneven illumination. B) The Incubot was requested to perform an imaging sweep of HeLa-GFP seeded culture wells every hour for 24 h. Representative images show the progression of a single region of a single FOV from 4 h post-seeding to 24 h post-seeding. Scale bar indicates 250 µm.

Tissue culture 6-well plates were seeded with HeLa-GFP cells and placed in the plate-holder of the *Incubot*. An area of 7x6 FOVs within each well was imaged. The ImageJ plugin “Grid/Collection Stitching” [28] was applied and established using the appropriate conditions for the order of images and ordered to compute overlap, with the resultant image saved (Fig. 16A.). Stitching was successful and generated a large area view with cellular resolution.

### Long-Term Imaging

HeLa-GFP seeding was repeated for a fresh 6-well plate which was then placed in the *Incubot* plate-holder. A single region of a single FOV was assessed over time, showing clear visibility of cells over the duration of the experiment (Fig. 16B.)

### Software Validation

#### Raspberry Pi Compatibility

The *Incubot* GUI (“IncubotGUI.py”) was designed to be used on a Raspberry Pi 3B + with Python 3.7.3. To date, no alternative equipment has been tested for compatibility with the *Incubot*, however it is theoretically possible to generate a new code on an alternative system.

The initial portion of python code is responsible for importing all the relevant dependencies for running of the full code, this is partitioned into a general section and a Kivy section. Kivy is a GUI designing module for python 3 which allows for generation of “apps” within a python environment [29]. Each button within the app is generated within Kivy and bound to a function. The functions vary based on the button, for example pressing the “6-Well” button will allow the raspberry pi to change the variables associated with plate type, such as number of wells, well diameter, and the [X,Y] coordinate locations for the default initial imaging of each well. This button, along with others, also commands Kivy to re-initialise the user interface, allowing for an updated screen view for greater ease of use. Of important note is that the GUI developed and distributed here was only tested on a monitor with aspect ratio of 4:3. Other display ratios may require some alterations to the code. The camera preview uses manually specified coordinate and size positions within the code, and so will not be applicable for other screen sizes. This can be adapted within the source python code where the camera preview dimensions are specified to orientate correctly for your specific screen size. The following dimensions should work more effectively for the camera preview on the touch screen: [96, 17, 263, 139). Unfortunately, we were unable to test the GUI with a raspberry pi touchscreen.

### Z-Axis Automated Focussing

Z-axis automated focussing was optimised using an image stack taken of low-density HeLa-GFP cells grown in monolayer conditions. A Z-stack with slice intervals of 0.2 mm was acquired using the *Incubot* (1680x1200, BMP format). Images were assessed for sharpness of edges using a Laplacian filter with, or without, a Gaussian filter or varying radius applied beforehand. The images were manually assessed for the slice with optimal focus, and then the output sharpness measurements were compared to determine which parameters allowed for correct selection of the most in-focus slice. Pure Laplacian filtering, and Laplacian filtering with a Gaussian pre-filtering (radius = 1) resulted in the selection of the correct image slice. Increasing radius of the Gaussian pre-filtering step (radius = 2, radius = 3) resulted in increasingly inaccurate assessment of optimal image slice. Thus, a Laplacian filter was applied to images with a Gaussian pre-filtering step using a radius of 1 pixel.

### Adaptability

A key benefit of open source hardware is the facilitation of adaptation and modification. We here discuss some of the potential modifications to this build that could enhance its utility for specific purposes.

### Optics

The build described here makes use of a Raspberry Pi camera system for image acquisition and Thorlabs optics components with a commercial objective lens. Each component in this system could easily be redesigned in numerous ways. The Thorlabs components may be replaceable using 3D printed components designed with well-designed threading. For example, the OpenFlexure microscope has already shown 3D printed optical components don’t pose a barrier to quality imaging [17], and could serve to replace our entire optics system. Any image acquisition equipment compatible with Raspberry Pi will work in place of the PiCamera used here and could further facilitate either cost-reduction (e.g. using a webcam sensor) or increased imaging ability/quality (e.g. using a high-quality sensor). The use of the open-source PiCameraX system (https://pypi.org/project/picamerax/) could also boost the imaging performance of this build. Many potential improvements are offered by this package, including lens shading correction, which could prove very useful for imaging quality. Incorporation of specific improvements would be up to individual users, but please note this would require significant alterations to the *IncubotGUI* source code.

### Maximum Plate Capacity

The area capable of being imaged was limited in our build by two key parameters: 1) the dimensions of the incubator, and 2) the range of the X and Y axes of the original Tronxy 3D printer. The dimensions of the incubator are fixed for any incubator, while the range of the printer can be extended. Recreational 3D printer users have increased the print areas for cheap printers using some easily accessible components [30]. For the Tronxy X1 printer used in our build, the X and Y axes could be extended using longer aluminium extrusion components to allow for a larger area capable of being imaged, which could facilitate the imaging of multiple plates simultaneously. While we utilise the Tronxy 3D printer’s full Z axis range, it is not essential for imaging. Therefore, adaptation of the Z axis to be shorter could increase stability of the build while not impacting the necessary range of the device.

### LED Assembly

We utilised cheap, readily available, 5 mm diameter LEDs for this build. It would be easy to replace the LEDs here with more sophisticated diodes, potentially with higher degrees of directionality, more specific wavelength emission, and controllable illumination power. A NeoPixel LED ring was originally tested with the *Incubot* and showed some success. A specific advantage of this system is the highly coordinated and specialised control of LED illumination, with RGBW illumination possible from multiple directions at a range of intensities. Issues may arise from the fact that the light was not directed towards the focal point of the optics, however this may be correctable through the addition of small silicone lenses to focus the LEDs to a desirable focal point. Adaptation by using LEDs with highly specific wavelength emission, in conjunction with the use of specific filters within the optics system, fluorescent imaging of a wide range of fluorophores is theoretically possible using this microscope.

### Concluding Remarks

The *Incubot* represents an addition to the current open source microscopy options available to research scientists. It has proven ability in white light and fluorescent imaging of live and fixed cell culture monolayer within a tissue culture incubator, maintaining stability and reliability. The low-cost of the build compared to commercial options and the open source nature of the microscope facilitate its accessibility to a wide range of potential users.

### Capabilities

• Long-term fluorescent or oblique imaging of samples
• Structurally stable within the incubator
• Allows for physiological imaging of live cell monolayers
• User-friendly GUI for easy-to-use microscopy system
• Individual cells and cell processes visible over time

### Limitations

• Upload to Dropbox cannot occur simultaneously with imaging
• Time for imaging may impede on some desired imaging experiments, requiring compromise from the researchers
• Unable to exit imaging experiment without hard shutdown
• While oblique illumination simplifies the process of fluorescent imaging, it does result in illumination that is not uniform across the field of view (Fig. 15B).

## CRediT authorship contribution statement

**George O.T. Merces:** Methodology, Software, Validation, Investigation, Writing - original draft, Visualization, Formal analysis. **Conor Kennedy:** Methodology, Investigation. **Blanca Lenoci:** Methodology, Investigation. **Emmanuel G. Reynaud:** Writing - review & editing, Investigation. **Niamh Burke:** Validation, Formal analysis. **Mark Pickering:** Supervision, Funding acquisition, Project administration, Resources, Writing - review & editing, Conceptualization, Investigation.
